# Imaging sensitive and drug-resistant bacterial infection with [^11^C]-trimethoprim

**DOI:** 10.1172/JCI156679

**Published:** 2022-09-15

**Authors:** Iris K. Lee, Daniel A. Jacome, Joshua K. Cho, Vincent Tu, Anthony J. Young, Tiffany Dominguez, Justin D. Northrup, Jean M. Etersque, Hsiaoju S. Lee, Andrew Ruff, Ouniol Aklilu, Kyle Bittinger, Laurel J. Glaser, Daniel Dorgan, Denis Hadjiliadis, Rahul M. Kohli, Robert H. Mach, David A. Mankoff, Robert K. Doot, Mark A. Sellmyer

**Affiliations:** 1 Department of Radiology and; 2 Department of Bioengineering, University of Pennsylvania, Philadelphia, Pennsylvania, USA.; 3 Department of Gastroenterology, Hepatology and Nutrition, Children’s Hospital of Philadelphia, Philadelphia, Pennsylvania, USA.; 4 Department of Biochemistry and Biophysics, University of Pennsylvania, Philadelphia, Pennsylvania, USA.; 5 Department of Pathology and Laboratory Medicine, Hospital of the University of Pennsylvania, Philadelphia, Pennsylvania, USA.; 6 Department of Medicine, Division of Allergy, Pulmonary, and Critical Care Medicine, and; 7 Department of Medicine, Division of Infectious Disease, University of Pennsylvania, Philadelphia, Pennsylvania, USA.

**Keywords:** Infectious disease, Bacterial infections, Diagnostic imaging

## Abstract

**BACKGROUND:**

Several molecular imaging strategies can identify bacterial infections in humans. PET affords the potential for sensitive infection detection deep within the body. Among PET-based approaches, antibiotic-based radiotracers, which often target key bacterial-specific enzymes, have considerable promise. One question for antibiotic radiotracers is whether antimicrobial resistance (AMR) reduces specific accumulation within bacteria, diminishing the predictive value of the diagnostic test.

**METHODS:**

Using a PET radiotracer based on the antibiotic trimethoprim (TMP), [^11^C]-TMP, we performed in vitro uptake studies in susceptible and drug-resistant bacterial strains and whole-genome sequencing (WGS) in selected strains to identify TMP resistance mechanisms. Next, we queried the NCBI database of annotated bacterial genomes for WT and resistant dihydrofolate reductase (DHFR) genes. Finally, we initiated a first-in-human protocol of [^11^C]-TMP in patients infected with both TMP-sensitive and TMP-resistant organisms to demonstrate the clinical feasibility of the tool.

**RESULTS:**

We observed robust [^11^C]-TMP uptake in our panel of TMP-sensitive and -resistant bacteria, noting relatively variable and decreased uptake in a few strains of *P*. *aeruginosa* and *E*. *coli*. WGS showed that the vast majority of clinically relevant bacteria harbor a WT copy of DHFR, targetable by [^11^C]-TMP, and that despite the AMR, these strains should be “imageable.” Clinical imaging of patients with [^11^C]-TMP demonstrated focal radiotracer uptake in areas of infectious lesions.

**CONCLUSION:**

This work highlights an approach to imaging bacterial infection in patients, which could affect our understanding of bacterial pathogenesis as well as our ability to better diagnose infections and monitor response to therapy.

**TRIAL REGISTRATION:**

ClinicalTrials.gov NCT03424525.

**FUNDING:**

Institute for Translational Medicine and Therapeutics, Burroughs Wellcome Fund, NIH Office of the Director Early Independence Award (DP5-OD26386), and University of Pennsylvania NIH T32 Radiology Research Training Grant (5T32EB004311-12).

## Graphical abstract

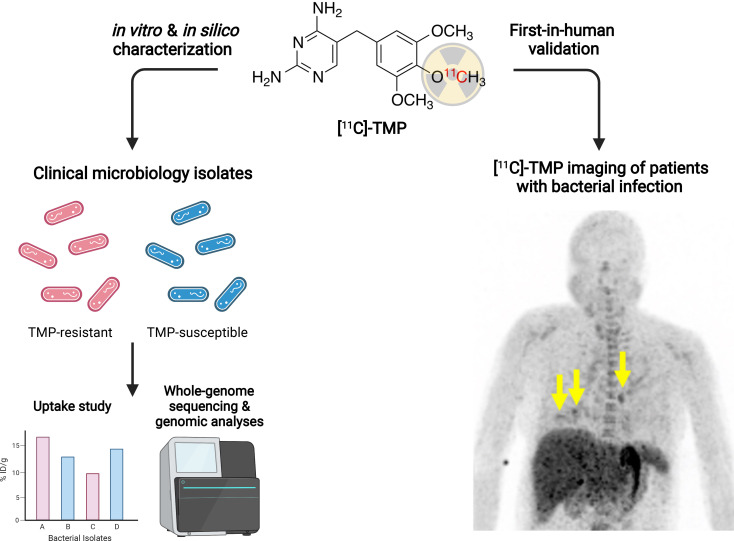

## Introduction

The ability to specifically detect and characterize a bacterial infection in a patient has been a long-sought goal for molecular imaging (1). The implications of such a technique are wide-ranging and could improve diagnosis of bacterial infection as well as allow quantitative monitoring of the effects of antimicrobial treatment. The current standards of biopsy and ex vivo microbial culture are hampered by contaminations, sampling limitations, variable sensitivity, potential for procedural complications, and delayed results (2). Thus, bacterial-specific radiotracers could have a positive effect on clinical practice and move the field of infection imaging beyond the current practices, which generally rely on nonspecific nuclear imaging approaches, such as radionuclide-tagged white blood cell, [^67^Ga]-citrate, and 2-deoxy-2-[18F]fluoro-D-glucose (FDG) scans (3, 4).

Several approaches have been pursued to develop bacterial-specific radiotracers. One method is targeting biochemical/metabolic transformations that are unique to bacterial biology. For example, promising advancements have been made in preclinical models using radiotracers, including [^18^F]-fluorodeoxy-sorbitol (FDS), [^11^C]-para-aminobenzoic acid, D-amino acids, and probes targeting the maltose transporter in bacteria (5–9). Only [^18^F]-FDS and [^11^C]-para-aminobenzoic acid have been reported in humans at this time (10, 11). Another strategy is to use radiolabeled antibiotics that can inhibit metabolic processes that are essential to the bacterial life cycle. We previously reported the development of [^11^C]- and [^18^F]-labeled trimethoprim-based (TMP-based) radiotracers and have demonstrated specificity for bacterial infection over other pathologies such as sterile inflammation (turpentine) and neoplasm (breast carcinoma) in rodent models (12, 13). TMP is generally thought to be a biologically inert and non-toxic in human cells at low concentrations. It affects nucleotide metabolism via competitive binding of bacterial dihydrofolate reductase (DHFR), with 10,000-fold selectivity over the mammalian enzyme (14). However, antibiotic radiotracers, such as TMP, have raised concerns that imaging antibiotic-resistant bacteria may be challenging. Furthermore, bacterial infection radiotracers need to maintain high uptake across a broad spectrum of bacterial species (e.g., targeting both Gram-positive and Gram-negative species) to produce favorable positive and negative predictive test characteristics when the causative organism is unknown (15).

Here, using previously tested laboratory bacterial strains and newly acquired, drug-resistant clinical isolates, we performed dose-response assays with TMP. We then tested uptake of [^11^C]-TMP in these pathogenic species to determine whether TMP resistance by itself is a critical factor for the “imageability” of the bacterial species. In addition, we performed whole-genome sequencing (WGS) of a subset of the clinical isolates to identify the number of DHFR genes and to probe for other resistance mechanisms present in these strains. To extend our WGS results, we used the NCBI database of annotated bacterial whole genomes and assessed the top 19 pathogenic bacterial species for the presence of WT and mutant DHFR enzymes. With evidence of the ability to image both TMP-susceptible and -resistant strains, we report the first-in-human feasibility study of bacterial infection imaging with [^11^C]-TMP, with a focus on lung and bone infections. We discuss cases demonstrating the biodistribution and specificity of [^11^C]-TMP uptake in humans and include instances when antibiotic treatments were administered to highlight the potential of [^11^C]-TMP to add diagnostic information in different clinical scenarios of bacterial infection.

## Results

**Figure 1:**
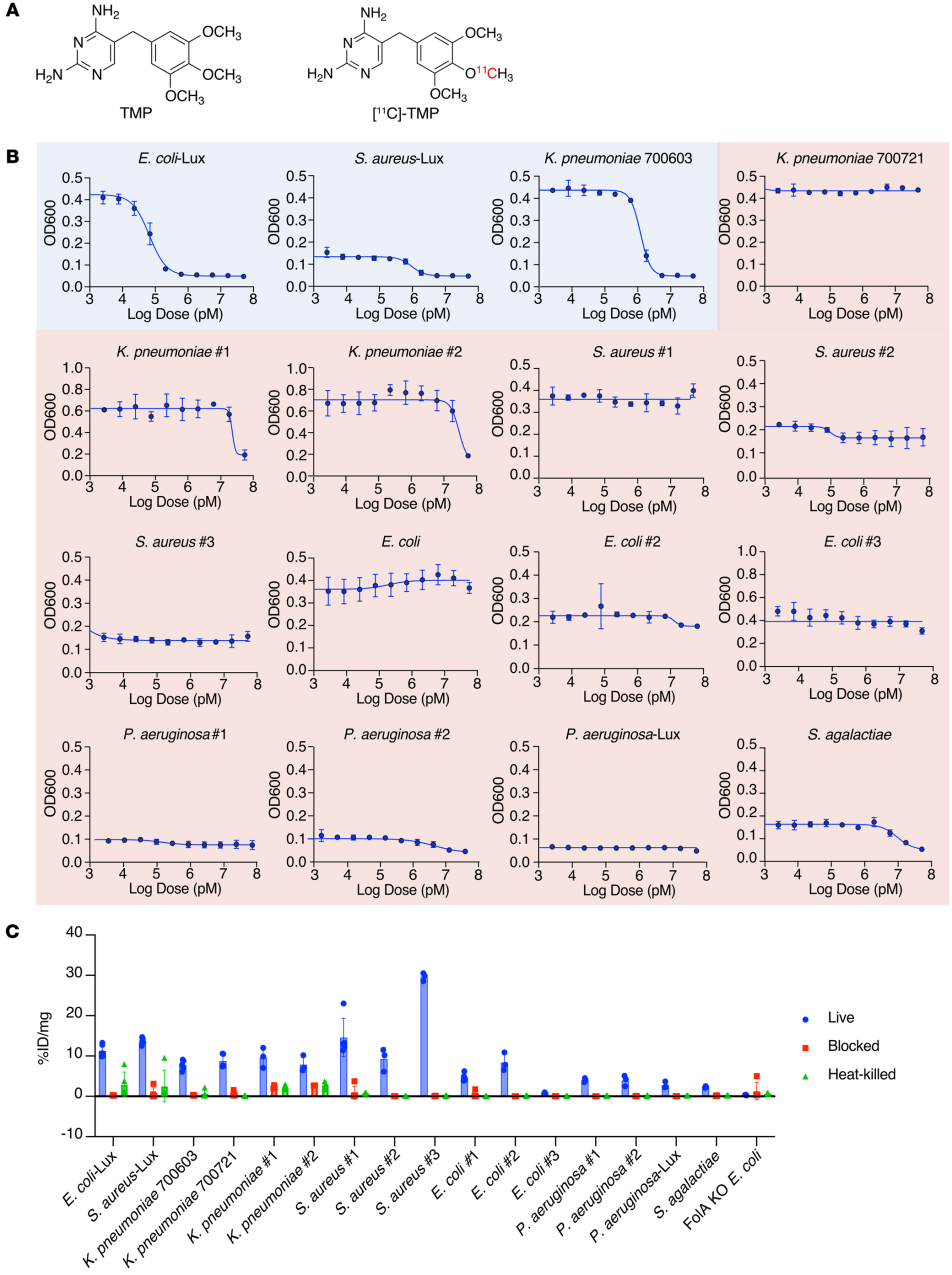
Structure of [^11^C]-TMP and in vitro TMP dose-response assays of different bacterial strains. (**A**) Structures of trimethoprim (TMP) and [^11^C]-TMP. (**B**) TMP dose-response assay on bacterial strains. OD600 measurement was taken following a 6-hour incubation of different bacterial strains with TMP. The susceptibility or resistance of a bacterial strain to TMP is color-coded based on the IC_50_ and minimum inhibitory concentration (MIC). Blue indicates susceptible bacteria, and red indicates resistant bacteria. *n* = 3; data represent mean ± SD. (**C**) Representative [^11^C]-TMP uptake in bacterial cultures after a 30-minute incubation at 37°C. *n* = 3–5; data represent mean ± SD. The experiment was repeated a total of 2–3 times for biological replicates.

[^11^C]-TMP was synthesized as previously reported (Figure 1A; ref. 13). We have tested TMP-sensitive bacterial strains in animal models (12), and thus, TMP-resistant clinical isolates of the same species were acquired (details included in Table 1). All strains were classified as susceptible or resistant by their TMP minimum inhibitory concentration (MIC) (Supplemental Figure 1A; supplemental material available online with this article; https://doi.org/10.1172/JCI156679DS1). MIC cutoffs were based on CLSI M100 break points (see *MIC assays* in Methods). To further characterize the susceptibility or resistance of our bacterial panel to TMP, we performed dose-response assays. Each bacterial strain was incubated with varying concentrations of TMP (0–50 μM) for 6 hours, and growth endpoints were recorded (Figure 1B and Supplemental Figure 1B for luminescence data). IC_50_ values are summarized in Supplemental Figure 1C.

[^11^C]-TMP uptake was tested in both TMP-susceptible and TMP-resistant bacteria by incubating the strains for 30 minutes with the radiotracer. Heat-killed or excess unlabeled TMP-blocked conditions served as controls. A DHFR-knockout strain of *Escherichia coli* (*E*. *coli*) K549 (ΔfolA) was tested as an additional negative control. Substantial increased uptake was observed across most of the strains, regardless of their susceptibility to TMP (Figure 1C). For comparison, uptake values were also normalized to the uptake values of the corresponding strain’s blocked control for comparison across species, which showed between approximately 5- and 500-fold increased uptake (Supplemental Figure 2). *E*. *coli* no. 3 and strains of *Pseudomonas aeruginosa* exhibited a relatively low level of uptake, and importantly, DHFR-knockout *E*. *coli* K549 (ΔfolA) showed no radiotracer retention. This varied yet maintained [^11^C]-TMP uptake in resistant strains suggested that TMP resistance was not simply the mutation of the TMP binding site of the endogenous DHFR gene and that further study was warranted.

Resistance to TMP is known to be mediated by altered gene regulation, leading to increased translation of the DHFR enzyme as well as horizontal gene transfer of mutant DHFRs, to which TMP binds poorly (16). To identify the resistance mechanism(s) present in our panel of bacteria, we performed WGS on the following TMP-resistant subset of strains; they were chosen from an initial panel of bacterial strains prior to uptake studies: *E*. *coli* no. 1, *Klebsiella pneumoniae* 700721, *Streptococcus agalactiae*, *P*. *aeruginosa* no. 1, and *Staphylococcus aureus* no. 1. The genomes were assembled using an A-Bruijn assembler, and the completeness of the genomes was assessed using sets of single-copy genes within a phylogenetic lineage (17, 18). Quality control approaches of WGS analysis are summarized in Supplemental Figures 3 and 4. Following quality control, predicted DHFR ORFs from the assembled genomes of all 5 strains were individually investigated using the NCBI’s BLASTp database and a literature search (Table 2 and Supplemental Figure 5). Based on the analysis, *S*. *aureus* and *E*. *coli* each contained 2 different DHFR genes. In both cases, the first gene was a resistant DHFR enzyme termed *DfrA*, which mediates TMP resistance (19, 20). The second gene was the canonical WT DHFR for the strain that is known to bind TMP. *K*. *pneumoniae* similarly carried a WT DHFR known to bind TMP (21) as well as a DHFR gene, whose TMP binding, to our knowledge, is uncharacterized to date. However, only 1 DHFR ORF was found in *P*. *aeruginosa*. UniProt and NCBI databases suggest this is the native DHFR gene in *P*. *aeruginosa*. It has been shown that drug-resistant strains of *P*. *aeruginosa* express export pumps that confer multidrug resistance to TMP and other antibiotics (22, 23), which was apparent in our dose-response assays (Figure 1B and Supplemental Figure 1, B and C). Of note, previous reports showed that when drug-susceptible and -resistant strains of *P*. *aeruginosa* were lysed, TMP inhibited the catalytic activity of DHFR of all strains with equal potency (24), suggesting that *P*. *aeruginosa* DHFR may still bind to TMP radiotracers. For *S*. *agalactiae,* only 1 DHFR was found in the genome, with no reports on whether it confers resistance.

**Figure 2:**
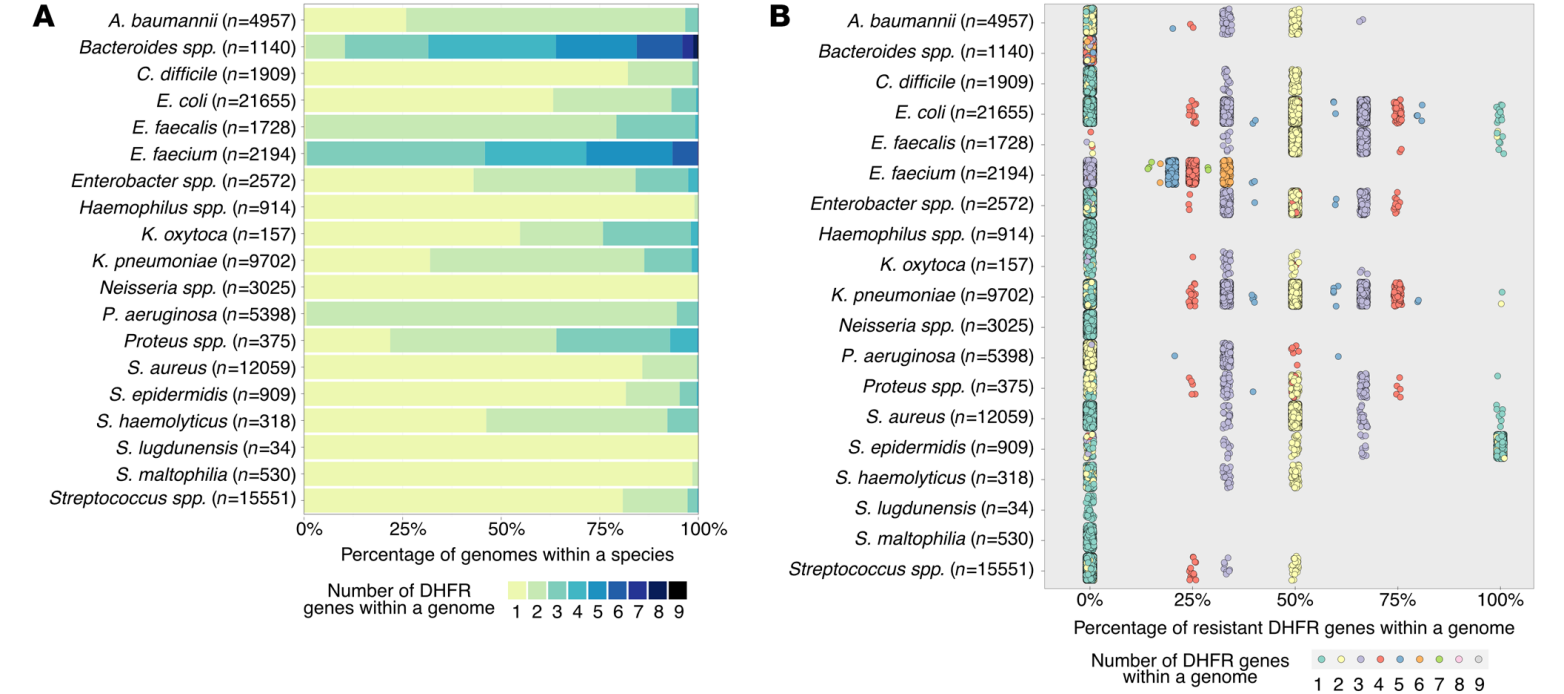
Bioinformatic analysis of clinically relevant bacteria. (**A**) Proportion of clinically relevant bacterial strains from the NCBI RefSeq database, with the indicated number of DHFR genes per genome. (**B**) Resistance characterization of DHFR genes in relevant bacterial strains from the NCBI RefSeq database. Each dot represents a strain.

It has been shown that DHFR redundancy can be a common feature of clinically relevant bacteria (25). Given the potential implications for clinical imaging with TMP radiotracers and our observations from WGS of several strains that maintained TMP uptake despite resistance, we broadly surveyed the DHFR genes in the NCBI RefSeq deposited genomes of the top 19 most clinically relevant bacterial pathogens (Figure 2A). We found that there is high heterogeneity both between and within species regarding the number of DHFRs carried. For example, the 5 most common bacterial pathogens responsible for health care–associated infections are *E*. *coli*, *S*. *aureus*, *K*. *pneumoniae, P*. *aeruginosa*, and *Enterococcus faecalis* (26). These strains on average carry 1.44, 1.14, 1.84, 2.05, and 2.21 DHFRs per genome, respectively. The percentage of resistance-conferring DHFR genes was calculated using gene annotation. We found that there is almost always a nonresistant DHFR within these pathogenic species. In fact, only 0.56% of strains exclusively carried DHFRs that confer TMP resistance, a total of 742 of 132,878 strains assessed. Moreover, there is an expected pattern relating the number of total DHFR genes per genome and the proportion of those genes that are TMP resistant (Figure 2B and Supplemental Figures 6 and 7). Taking *E*. *coli* as an example, when a strain bears only 1 DHFR, that single gene is not resistant in almost all cases. Conversely, when a strain bears 2 DHFRs, typically 1 of the 2 is resistant (50%). Furthermore, when strains bear 3 DHFRs, typically either 1 or 2 DHFRs (33% and 67%) confer resistance. The pattern holds for 4 and 5 DHFRs and so on (Supplemental Figure 6). Thus, it is reasonable to assume that [^11^C]-TMP has the potential to image TMP-resistant infections in diverse clinical settings, with caveats that rare bacterial strains that have a lone copy of a TMP-resistant DHFR gene or otherwise limited uptake.

**Figure 3:**
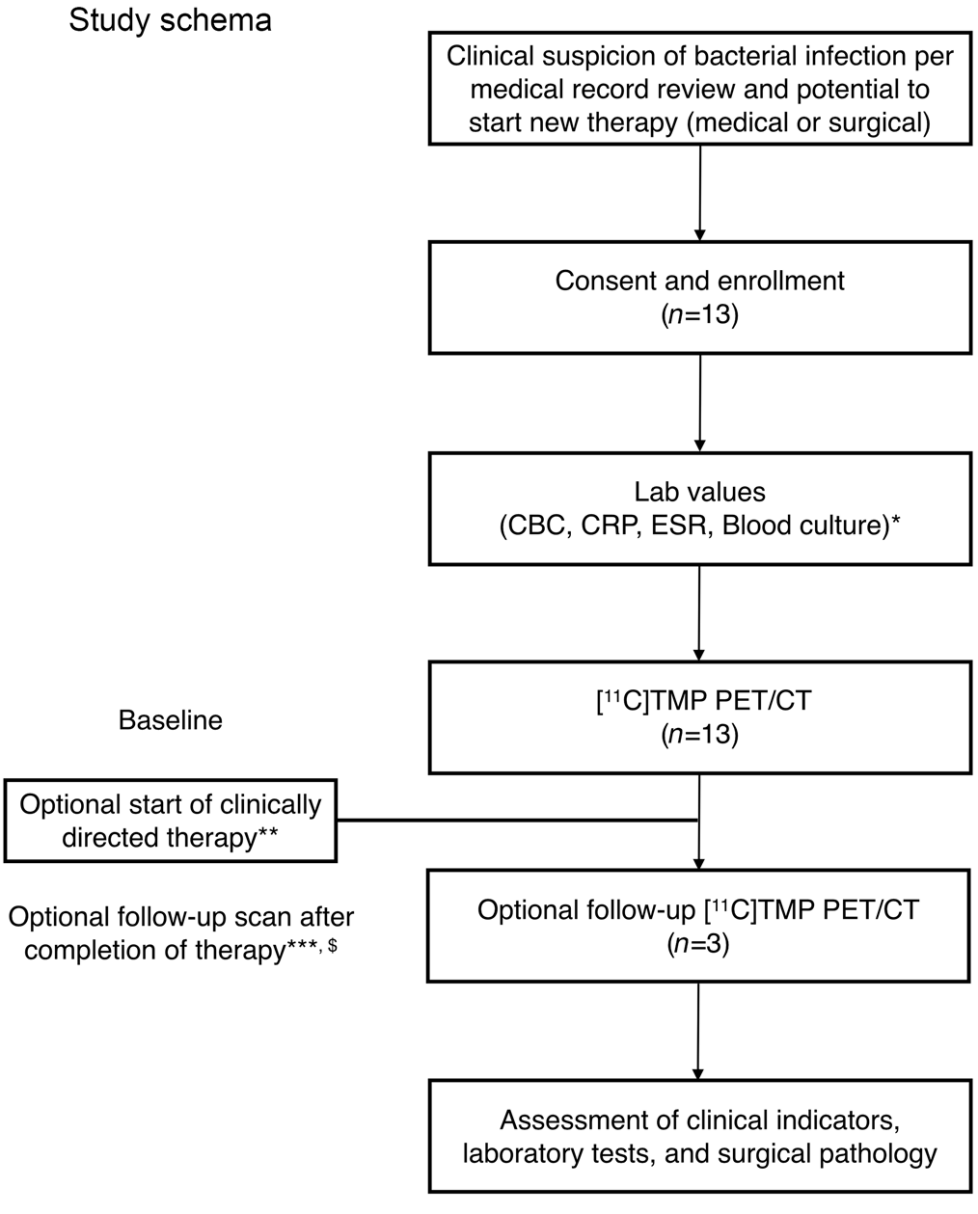
CONSORT diagram of the [^11^C]-TMP study. *Laboratory test results may have been collected from the medical record if they were completed within 30 days of screening; in these cases, they were not repeated for the purposes of this study. Refusal of labs did not preclude a patient from the study. **Surgical or systemic therapy was started if clinically indicated at the judgment of treating physicians. ***For patients who received systemic antibiotic therapy, this may have been within 1 week of therapy cessation. For patients who received surgical management, this may have occurred within 3–6 weeks after surgery, as clinical appropriate. Given that some patients are on chronic antibiotics, this scan may have occurred after completion of alternative or more intensive antibiotic therapy. ^$^In some cases, and at the discretion of the investigator, the patient may have been scanned while on i.v. antibiotics.

**Figure 4:**
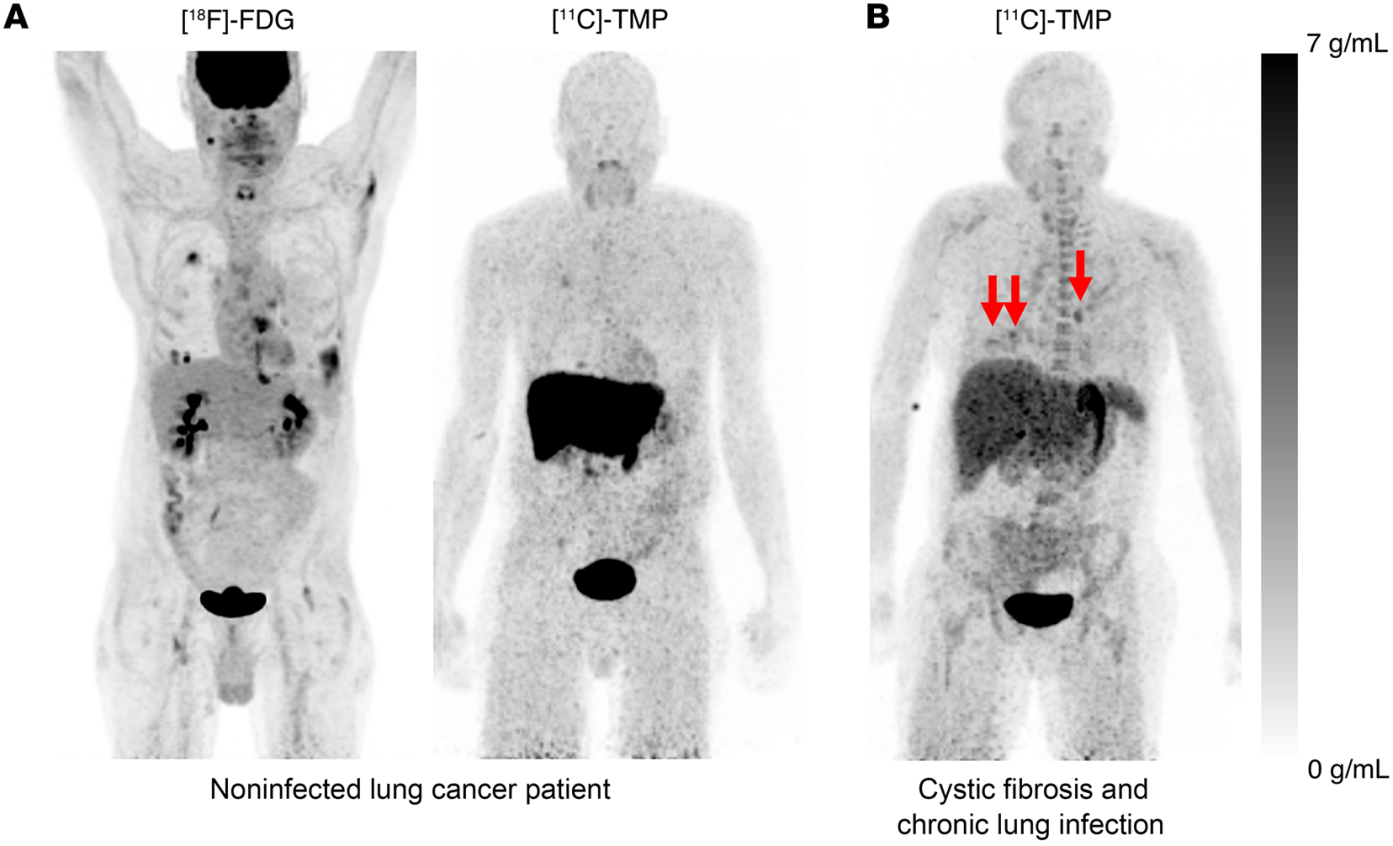
Biodistribution of [^18^F]-FDG versus [^11^C]-TMP in a patient with lung cancer and biodistribution in a patient with underlying chronic lung disease. (**A**) The image on the left shows a 64-year-old man with known lung adenocarcinoma who underwent a [^18^F]-FDG (549 MBq) and then a [^11^C]-TMP (563 MBq) PET/CT 2 days later. The [^18^F]-FDG image was acquired starting 71 minutes after injection. Whole-body maximum intensity projection (MIP) images demonstrate the difference in biodistribution of the tracers. In the lungs, [^18^F]-FDG is taken up both by metabolically active tumor and inflammatory cells, whereas [^11^C]-TMP is not. The image on the right shows a comparison MIP image of a patient with cystic fibrosis and chronic lung infections who underwent a [^11^C]-TMP PET/CT (780 MBq). The image was acquired starting 78 minutes after injection. PET images are scaled at 0–7 g/mL SUV. (**B**) [^11^C]-TMP imaging of a 44-year-old woman with several foci of infection in the chest (red arrows). Other sites of signal include the liver, the kidneys, red bone marrow, and the stomach. PET images are scaled at 0–7 g/mL SUV.

Based on our preclinical evaluation of TMP radiotracers in animal models of infection (12), and these promising in vitro data, we developed a clinical protocol to assess the biodistribution of [^11^C]-TMP in humans. A CONSORT diagram summarizing patient enrollment and study flow is presented in Figure 3. Inclusion/exclusion criteria and a demographics table are included in Supplemental Figure 8. Patients were consented for the investigational protocol (NCT03424525), and several case examples of feasibility are presented here. First, a biodistribution study was performed in a 64-year-old male that was being surveilled with [^18^F]-FDG PET/CT for metastatic lung adenocarcinoma (Figure 4A, left). [^18^F]-FDG, a fluorinated glucose derivative, is often used in patients with cancer to stage or monitor therapy, as tumors are highly metabolically active. The diagnostic challenge with [^18^F]-FDG is that both cancer and infection/inflammation often show elevated levels of FDG uptake. The patient had several sites of [^18^F]-FDG avid lung and distal metastases that maintained a low level of background uptake on the [^11^C]-TMP scan, suggesting that tumor metabolism does not confound [^11^C]-TMP uptake (Figure 4A, right, and Supplemental Figures 9–11). Conversely, in a patient with a known chronic lung infection, a 44-year-old woman with cystic fibrosis (CF), there were multiple foci of radiotracer uptake in the lungs corresponding with areas of multifocal pneumonia (Figure 4B). Notable sites of [^11^C]-TMP metabolism and excretion in patients included the liver, kidneys, and bladder, while many tissues that could be potential sites of infection showed very low background uptake. Radiotracer uptake was noted in vertebral bodies and proximal long bones for patients with metabolically active marrow (e.g., young women), and the time activity curves of select vertebral bodies were calculated for several patients showing that the [^11^C]-TMP uptake in bones does not increase over time (Supplemental Figure 12).

**Figure 5:**
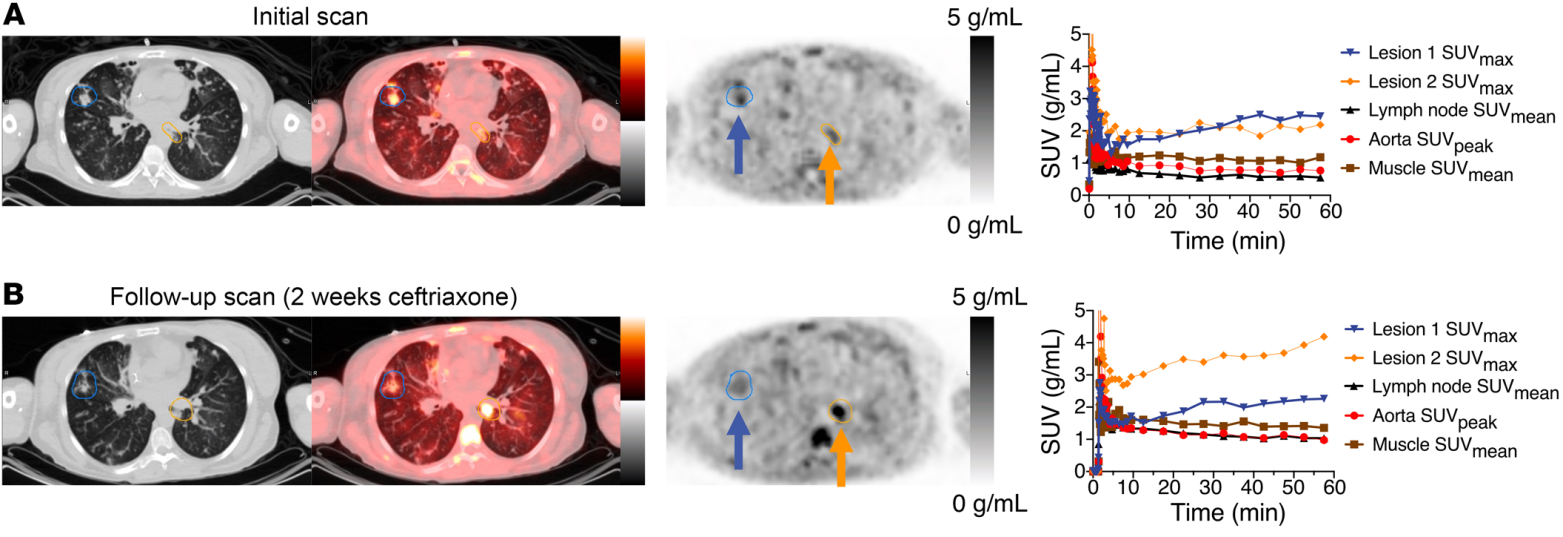
Time activity curves and bacterial heterogeneity. (**A** and **B**) [^11^C]-TMP PET/CT images of a 44-year-old woman with an acute exacerbation of cystic fibrosis (**A**) before and (**B**) after treatment with 2 weeks of i.v. ceftriaxone. Regions of interest were drawn around 2 separate pulmonary airspace opacities. In addition, reference regions were also drawn around a reference lymph node and paraspinal musculature and within the aorta. Comparing PET/CT images before and after treatment, the visible changes in relative [^11^C]-TMP uptake in lesion 1 compared with lesion 2 demonstrate the bacterial heterogeneity and an apparent new infection with *S*. *aureus* based on sputum cultures. The patient received 487 MBq and 780 MBq of [^11^C]-TMP at the first and second time points, respectively. PET images are scaled at 0–5 g/mL SUV and CT images are scaled at –1,024 to +300 HU.

Analyses of the lesions in the patient with CF shown in Figure 4B had varying time activity curves. Several lesions showed increased uptake over time (Figure 5A). This uptake contrasted with the washout kinetics of the muscle, lymph nodes, and aorta (blood pool) measurements in maximum standardized uptake values (SUV_mean_) (given that partial volume effects are less of a concern in these reference tissues). Sputum cultures around the time of initial imaging from this patient grew cephalosporin-susceptible but TMP-resistant *E*. *coli* (in addition to pan-resistant *Achromobacter*). She was placed on i.v. ceftriaxone for 2 weeks, and a follow-up [^11^C]-TMP scan at the end of treatment (4 weeks after the first PET/CT) showed improvement in the right lower lobe foci on both PET and CT imaging. However, a new focus developed in the para-aortic medial left lower lobe of the lung of the patient. This focus has an SUV_max_ of 4.0 (Figure 5B). A repeat sputum culture at the time of follow-up imaging grew methicillin- and TMP-sensitive *S*. *aureus* in addition to her chronic *Achromobacter*. Thus, the radiotracer continued to show increased uptake in the setting of positive sputum cultures and despite antibiotic treatment. The patient’s lung function continued to worsen, and she later went on to receive a bilateral lung transplant.

**Figure 6:**
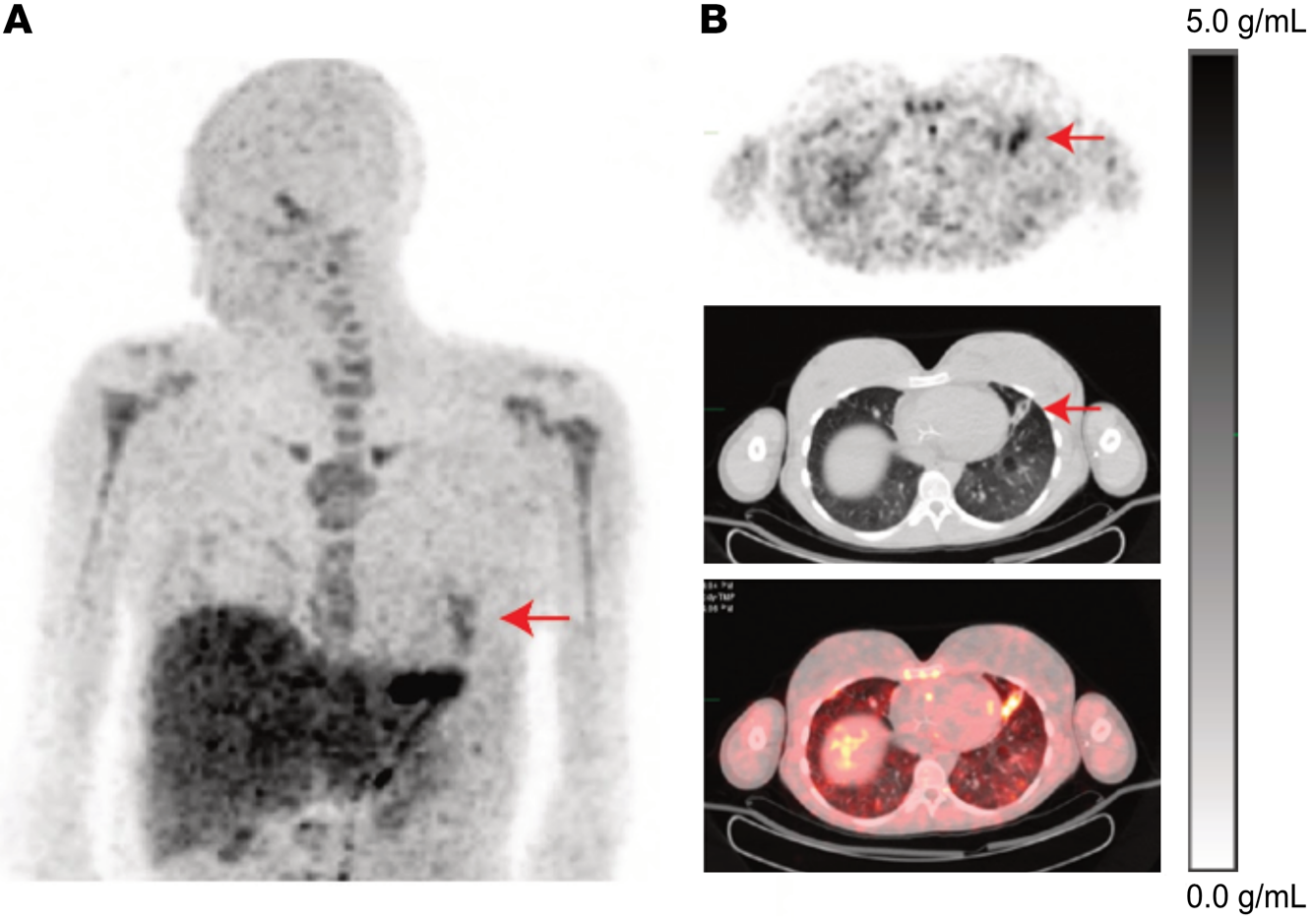
Acute exacerbation of cystic fibrosis. (**A**) A 64-year-old man with known lung adenocarcinoma underwent a [^18^F]-FDG (549 MBq) and then a [^11^C]-TMP (563 MBq) PET/CT 2 days later. The [^18^F]-FDG image was acquired starting 71 minutes after injections. Whole-body maximum intensity projection (MIP) images demonstrate the difference in biodistribution of the tracers. In the lungs, [^18^F]-FDG is taken up both by metabolically active tumor and inflammatory cells, whereas [^11^C]-TMP is not. (**B**) A comparison MIP image of a 44-year-old woman with cystic fibrosis and chronic lung infections, who underwent a [^11^C]-TMP PET/CT (780 MBq). The image was acquired starting 78 minutes after injection. The PET images show several foci of infection in the chest (red arrows). Other sites of signal include the liver, the kidneys, red bone marrow, and the stomach. PET images are scalled 0-7 g/mL SUV.

In a companion case, a 21-year-old female patient who also had CF was scanned 2 days into an inpatient course of i.v. antibiotics. Her scan showed an area of left lung consolidation with increased [^11^C]-TMP uptake (Figure 6). This patient’s lung function and symptoms improved on antibiotics, and she was discharged from the hospital without a follow-up [^11^C]-TMP PET/CT scan.

**Figure 7:**
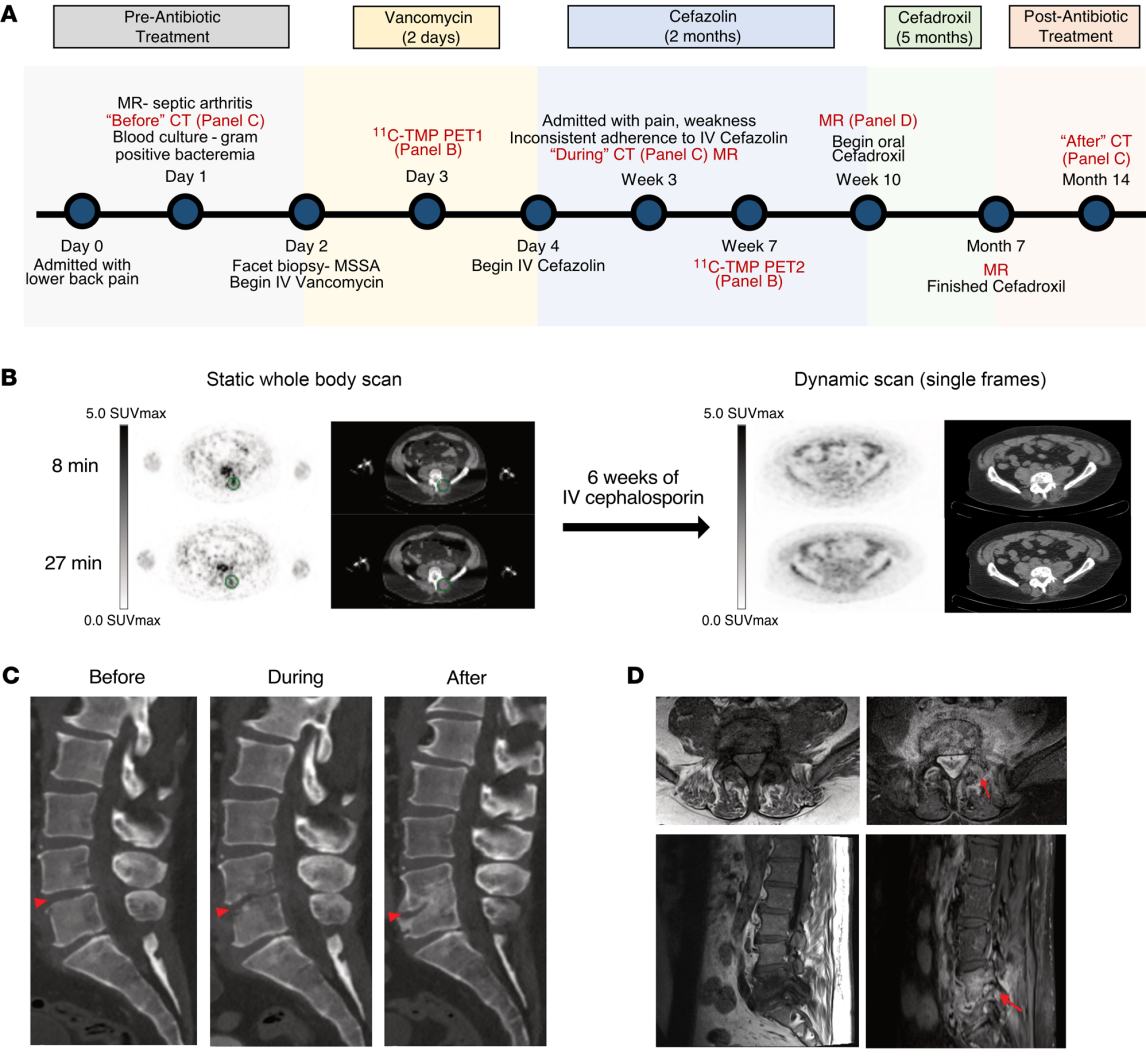
Biopsy proven discitis osteomyelitis treated with antibiotics. A 55-year-old man with clinically suspected lumbar discitis osteomyelitis was scanned with [^11^C]-TMP PET/CT at the initiation of empiric antibiotic therapy and after 6 more weeks of targeted antibiotic therapy. (**A**) Time line of patient disease sequelae and imaging. (**B**) Axial PET/CT images show a clear site of asymmetric [^11^C]-TMP uptake in the left L4–L5 facet at the start of therapy and lack of uptake after 6 weeks of i.v. treatment. Facet biopsy of the left L4–L5 facet grew methicillin-sensitive *S*. *aureus*. Note: the patient received different doses of [^11^C]-TMP, 129 MBq at the first time point and 672 MBq at the second time point; thus, the image quality was noisier at the first time point. PET images are scaled at 0–5 SUV_max_. (**C**) The temporal morphologic sequelae of discitis osteomyelitis are demonstrated by sagittal CT images before, during, and after treatment. (**D**) In contrast to the PET/CT images, the gadolinium-enhanced MRI images of the patient at 10 weeks after therapy continue to demonstrate marrow replacement and contrast enhancement, findings that are nonspecific for active infection versus continued inflammation.

Finally, a 55-year-old male patient with biopsy-proven methicillin-sensitive *S*. *aureus* (MSSA) discitis osteomyelitis, with contiguous involvement of the left L4–L5 facet and surrounding musculature, was scanned with [^11^C]-TMP (Figure 7A). The patient underwent different scanning protocols (biodistribution versus kinetic) on different days (early and after treatment), with differences in radiotracer dosage and image acquisition timing. To optimally compare the two studies, the treatment follow-up dynamic scan images were reconstructed at 8 and 27 minutes after injection to match the initial biodistribution imaging time points (Figure 7B and Supplemental Figure 14). After 6 weeks of i.v. cefazolin therapy, there was a resolution of radiotracer uptake at the left L4–L5 facet. These functional imaging findings preceded the anatomic sequelae of infection and remodeling seen on CT during the treatment and after treatment interval (Figure 7C). Despite resolution of radiotracer uptake and no subsequent clinical recrudescence of infection, an MRI scan after therapy suggested the potential interval worsening of discitis osteomyelitis (Figure 7D).

## Discussion

Although bacterial infections often are treated effectively by antibiotic therapy, the incidence of multidrug-resistant strains continues to rise and has a profound effect on modern medical care. Our diagnostic armamentarium for bacterial infections needs to be improved, and molecular imaging can greatly contribute to the effort. Nuclear imaging, especially with anatomic correlation via CT or MRI, has the sensitivity to detect infections in humans. Here, we described an assessment of a TMP radiotracer in antibiotic-susceptible and -resistant bacterial strains, cataloged many of the most pathogenic strains of bacteria with respect to their potential imageability, and provided several case examples of first-in-human [^11^C]-TMP imaging.

We sought to understand whether antibiotic resistance would abrogate antibiotic tracer uptake. We found that drug-resistant bacterial species had similar uptake of [^11^C]-TMP as nonresistant species, suggesting that antibiotic resistance alone was not a critical feature affecting radiotracer uptake. We observed relatively low tracer absolute accumulation in *E*. *coli* no. 3 and strains of *P*. *aeruginosa* (Figure 1C). Given that [^11^C]-TMP uptake was varied yet maintained above background (Supplemental Figure 2) in the resistant strains, we performed WGS and identified that 3 resistant strains carried a second DHFR gene, with the WT copy preserved. Taken together, these results suggested that the maintenance of at least 1 WT copy of DHFR is a critical component needed to maintain imageability.

Next, we cataloged the annotated DHFR genes from 19 bacterial species that are common causes of pathologic human infections. Leveraging decades of research in antibiotic resistance mechanisms and genomic data (27, 28), we found that RefSeq-deposited strains carried anywhere from 1 to 9 copies of DHFR (Figure 2B). Outliers among the species included *E*. *faecalis* and *P*. *aeruginosa*, where almost all strains analyzed contained multiple copies of DHFR, whereas *Staphylococcus lugdunensis*, *Haemophilus spp*., and *Stenotrophomonas maltophilia* mostly contained a single copy of DHFR. Bacteria such as *Bacteroides spp*. and *E*. *faecium* had the most redundant copies of DHFR. Almost all strains contained at least 1 copy of WT DHFR. For example, only 742 strains of 132,878 strains assessed contained resistant DHFR copies only. That equals 0.56% of potential pathogenic bacteria included in our search, suggesting a strong potential for [^11^C]-TMP to detect many different bacterial strains in patients, assuming other criteria, such as bacterial density and background tissue uptake, are not limiting. Interestingly, we see strong in vitro uptake in bacteria expressing drug export pumps. Several of these bacteria (*S*. *aureus* no. 1, *K*. *pneumoniae 700721*, *E*. *coli* no. 1, *P*. *aeruginosa* no. 1) that underwent WGS expressed drug export pumps (Supplemental Figure 13) yet showed [^11^C]-TMP uptake to be maintained. It is likely that the intracellular concentration of radiotracer needed to detect and/or image a strain can be less than the amount of drug needed to exert antimicrobial effects. For example, *E*. *coli* bacterial strains that express an export pump (TolC) have shown accumulation of fluorescent TMP to a comparable level as an efflux pump-deficient strain (ΔtolC) (29). While the mechanism of this preserved accumulation needs to be further validated through future studies, the presence of DHFR as a molecular sink, in addition to an active influx system that exceeds the rate of efflux or Donnan potential, has been proposed as a possible mechanism behind the observed cellular accumulation (30, 31). These in vitro assays and genomic data set suggest that antimicrobial tracer binding is not categorically related to strain antibiotic resistance or its mechanism of resistance. Future uptake studies using knockout or single antimicrobial resistance (AMR) gene addition comparator strains that use different AMR mechanisms are being pursued.

To understand the background uptake in terms of biodistribution and present early experience with [^11^C]-TMP in patients with suspected bacterial infection, we developed a first-in-human clinical protocol. The biodistribution of [^11^C]-TMP is naturally different from that of the commonly used metabolic radiotracer [^18^F]-FDG (Figure 4). Due to the low background radiotracer uptake in many tissues (i.e., the lungs, muscles, brain, and vasculature), the sensitivity of [^11^C]-TMP to detect acute bacterial infection is promising. Organs with the most radiotracer uptake in noninfected patients were the liver, kidneys, and bladder, which are the expected organs of metabolism and excretion. Future studies including [^11^C]-TMP dosimetry and kinetic modeling are nearing completion and will yield more insight into the biodistribution imaging methodology of the tracer in patients.

TMP-based radiotracers could be useful for patients with chronic infections such as CF. In this patient population, the bacterial densities in the lungs could be monitored over the natural course of the disease and could be complementary to the more invasive (albeit specific) bronchoalveolar lavage (32). We found that patients with lung infections had increased uptake in some, but not all, of their lung lesions, as identified on CT imaging (Figures 4–6). Future studies could assess whether lesions that show greater uptake are more likely to be the cause of the patient’s active symptoms. Such uptake could be monitored over time and relative to antimicrobial therapies. Alternatively, [^11^C]-TMP could be used as an additional biomarker in concert with clinical symptoms, biochemical lab values, and pulmonary function tests to stratify patients that may be candidates for lung transplantation. For example, a patient with advanced CF showed the development of a new focus of [^11^C]-TMP uptake while also demonstrating a new MSSA bacterial lung infection on sputum culture (Figure 5). One lesion showed increased uptake on a time activity curve following a course of antibiotic treatment, suggesting continued localization of the tracer to the infection (Figure 5B). This patient went on to receive a bilateral lung transplant because of her recurrent infections and poor pulmonary function.

Finally, we present a case of L4–L5 vertebral discitis osteomyelitis with a biopsy and bone culture that grew MSSA. The uptake that was associated with the facet and nearby soft tissue resolved after 6 weeks of i.v. antibiotic therapy, comparable to the lung infection cases, suggesting the potential of such bacterial imaging radiotracers to monitor infection treatment (Figure 7 and Supplemental Figure 14). The caveats of this case include that the patient had a facet biopsy prior to the PET imaging, which could cause local inflammation itself, and the patient was already being treated with i.v. vancomycin, potentially damping live bacteria in situ. Bony changes apparent on CT were poor surrogates for active infection, and the enhancement on MRI suggested continued osteomyelitis long after the patient had completed antibiotics and the [^11^C]-TMP uptake had resolved. Although no biopsy to prove sterility occurred at the end of therapy, there was no further clinical recrudescence of bony infection for this patient.

There are several important limitations to our studies. One is in the number of bacterial strains tested in vitro. It is possible that other untested strains could have significantly lower uptake than our panel of bacteria and that such a low level of uptake would portend those strains to be more difficult to detect in vivo. Further studies in animals, especially in nonhuman primates, may be helpful to better characterize such thresholds, as rodents have different immune systems, metabolic rates, and imaging constraints. Another limitation is that hepatobiliary clearance of the radiotracer likely would affect detecting an infection in the liver. Moreover, patients who are actively receiving TMP therapy would not be candidates for imaging, given the competition of the radiotracer and the antibiotic for DHFR. Yet another limitation is that this first-in-human [^11^C]-TMP study has a small sample size. Focused clinical population protocols and increased numbers of patients are needed to further validate the sensitivity and specificity of this approach in a patient subset or by clinical indication. Furthermore, while sputum culture results before and after treatment of the patient with CF, presented in Figure 5, showed resolution of her *E*. *coli* infection following ceftriaxone treatment (Supplemental Figure 8), we were unable to distinguish which PET lesions were caused by her acute *E*. *coli* infection, a chronic colonizer *Achromobacter*, or *S*. *aureus,* which was newly present on a follow-up sputum culture at the time of scan 2. Future animal studies, where TMP-sensitive and -resistant bacteria are inoculated in the lungs, can be used to support these findings, as our previous animal models have focused on myositis (12). Finally, there was [^11^C]-TMP uptake at the early time points in metabolically active marrow, seen best in vertebral bodies and proximal long bones in young female patients (Figure 6A). This uptake, however, appears to plateau (Supplemental Figure 12). This finding is not surprising, as sustained therapeutic dosing with TMP is known to suppress hematopoietic activity. Given the relatively low affinity of TMP for human DHFR, it will be important to characterize whether delayed imaging using radiotracer derivatives with longer-lived isotopes will allow for more washout from bone marrow.

Our group has previously developed [^18^F]fluoropropyl-trimethoprim (FPTMP) and demonstrated its ability to image bacterial infection in rodents (12). Given the longer half-life of ^18^F compared with ^11^C, the use of [^18^F]-FPTMP may allow for greater sensitivity and lower overall background signal. However, [^11^C]-TMP is an isotopolog of TMP, and the structural similarities of [^11^C]-TMP to its parent antibiotic highlights a potential regulatory advantage of the compound over the fluorinated version. In addition, ^11^C radiotracers may be administered to patients prior to ^18^F tracers such as FDG for dual characterization of lesions, for example, in a patient with known cancer and indeterminate lung lesions. Besides ^18^F and ^11^C, other groups have reported ^99m^Tc-labeled TMP through chelation using 2,4-diaminopyrimidine of TMP (33). However, the SPECT inherently has a lower sensitivity compared with PET (34), and the chemical modification of the pyrimidine, which serves an important role in the binding of TMP to eDHFR, likely would hinder and reduce the binding of the radiotracer to its target (35).

In summary, we presented [^11^C]-TMP uptake in bacteria that are both sensitive and resistant to TMP, described the mechanism of TMP resistance in clinical isolates using WGS, and applied a bioinformatic approach to highlight the potential of radiolabeled TMP to image different pathogens, regardless of resistance status. We also demonstrated several case examples of patients with proven infections. Future studies describing the imaging methodology, kinetics, dosimetry, and additional patient examples are in progress, and the studies presented here lay the foundation for future work characterizing the sensitivity and specificity of the TMP radiotracer family for bacterial infections.

## Methods

### Bacterial reagents

Please refer to Table 1 for information on bacterial strains and their sources.

### [11C]-TMP synthesis

[^11^C]CO_2_ was produced by a ^14^N(p,α)^11^C reaction using an IBA Cyclone 18; [^11^C]CH_3_I was synthesized from this using a gas-phase module (GE Healthcare). [^11^C]CH_3_I was trapped in a mixture of TMP-OH (0.75 mg, 2.70 mmol) and 5 N NaOH aqueous solution (5.4 μL, 27 μmol) in DMF (500 μL) at room temperature. The reaction mixture was heated at 70°C for 5 minutes and diluted with high-performance liquid chromatography (HPLC) mobile phase (1.0 mL, 12% EtOH in 0.01 M phosphate buffer, pH = 3.0). The solution was then injected onto a HPLC equipped with a semipreparative column (Phenomenex Gemini 5 μ, C18 110Å, New Column 250 × 10 mm) and eluted with HPLC mobile phase as above at a flow rate of 3 mL/min. The desired fraction eluted at 10–12 minutes was collected and used for biologic evaluation without concentration. For specific activity determination, an aliquot of [^11^C]-TMP was injected onto an HPLC equipped with an analytical column (Agilent XDB-C18, 5 μ, 150 × 4.6 mm) and eluted with 15% CH_3_CN: 85% water with 0.1% TFA at a flow rate of 1 mL/min (*t_R_* = 5.1–5.2 min). Specific activity determinations were carried out by comparing the UV peak area (wavelength, 230 nm) of the desired radioactive peak with those of different concentrations of TMP by HPLC. An aliquot of [^11^C]-TMP was coinjected with TMP into an HPLC system to confirm its identity.

### Bacterial cell culture

Individual colonies were picked on Luria-Bertani (LB) plates with appropriate selection antibiotics from Penn Cell Center. For experiments, all bacterial cultures were inoculated in LB broth with appropriate antibiotic selection and were shaken at 300 rpm and 37°C overnight. For bioluminescent strains of bacteria, bioluminescence is not known to affect pathogenicity or drug uptake.

### In vitro assays

#### MIC assays.

MIC evaluation was performed using TMP MIC test strips (Lilofilchem). Bacterial strains were grown in LB overnight and plated on Mueller-Hinton agar at an inoculum density of 0.5 McFarland. The plates were grown for 18 to 24 hours at 37°C, and the degree of inhibition was read. Based on CLSI M100 break points (30th edition), a TMP MIC value of more than or equal to 4 was considered resistant.

#### Bacterial growth inhibition-dose response curves.

All bacteria strains were grown overnight to saturation. On the day of the experiment, TMP was prepared in LB broth and was serially diluted 3:1 on a 96-well plate (with no drug control). 10 μL of the overnight bacterial cultures was diluted in 20 mL of LB, and 10 μL of this diluted bacterial culture was added to each well. The plates were then incubated for 6 hours at 37°C while shaking at 180 rpm before measuring OD600. Viability curves were plotted and analyzed on GraphPad Prism to determine an IC_50_.

#### [^11^C]-TMP uptake assays.

All bacteria strains were grown overnight to saturation. On the day of the uptake experiment, OD600 of the bacteria was measured and used to determine the number of CFU. The cultures were sedimented by centrifuging at 3,000*g* and were resuspended at a concentration of 5 × 10^9^ CFU/mL in LB broth. Three, 5 mL aliquots of each strain were prepared in tubes labeled as live, blocked, or heat killed. Heat-killed aliquots were then heated at 95°C for 45 minutes with intermittent vortexing of the samples every 15–20 minutes, while live and blocked bacteria were placed on ice.

Cold, unlabeled TMP was added to the blocked aliquots to a final concentration of 50 μM. A radiotracer dose of 5 × 10^6^ cpm was added to all aliquots of bacterial strains; the strains were incubated with the added radiotracer dose at 37°C for 30 minutes. Following incubation, the cultures were centrifuged at 3,000*g* and washed twice with ice-cold PBS. After the second PBS wash, the bacteria were again sedimented but then resuspended in 1 mL PBS and split into 5 technical replicates of 200 μL. Radiotracer uptake was measured on Gamma Counter (Perkin Elmer) with decay correction.

Once the radiotracer had decayed (10 half-lives), a Lowry assay (Thermo Scientific) was performed on each strain of bacteria to determine protein concentration. This protein concentration was used to normalize the radiotracer uptake to milligram of protein. All analysis was performed using GraphPad Prism 9.

### WGS for identification of TMP resistance mechanisms

Bacterial cultures of TMP-resistant strains (*E*. *coli* no. 1*,* *S*. *aureus* no. 1*,* *K*. *pneumoniae* 700721, *P*. *aeruginosa* no. 1*,* and *S*. *agalactiae*) were inoculated in 5 mL LB broth and shaken at 300 rpm and 37°C overnight. To prepare for WGS, 1 μL of the overnight bacterial cultures were diluted in 100 μL PBS.

DNA from cultured samples were isolated with the Qiagen DNeasy PowerSoil kit, and libraries were generated using the Nextera Flex Library Prep kit and sequenced on the Illumina HiSeq 2500 using 2 × 125 bp chemistry. Illumina sequencing reads were demultiplexed and quality filtered using the default settings of Trimmomatic (36), and adapters were trimmed from sequences with Cutadapt software (37). Low complexity sequences were masked using Komplexity (https://github.com/eclarke/komplexity/commit/d39bff9) with a normalized complexity score of less than 0.55. Reads that mapped to a human reference sequence (Genome Reference Consortium Human Build 38 [GRCh38]) were identified using bwa (38), and reads with more than 60% of the read fraction mapping to GRCh38 or with a percentage identity of more than 50% were removed. This produced a mean of 1.02 million host-filtered, quality-controlled reads per sample. SPAdes 3.14 (17) was used for de novo assembly of the host-filtered, quality-controlled short reads. The quality of the assembled genomes was assessed using CheckM v1.1.2 (18) and Anvi’o v6.2 (39) for completion and contamination. ORFs of the assembled genomes were identified using Prodigal v2.6.3 (40) and blasted against the Comprehensive Antibiotic Resistance Database v1.1.7 (41) and a manually curated list of DHFR genes. Pileup analysis was performed by aligning reads onto the assembled and reference WT DHFR genes using Bowtie2 (42) and counting the frequency of variants using dnapy (https://github.com/sherrillmix/dnapy/commit/e0435c2).

### Survey of DHFR genes in RefSeq-deposited genomes

The protein fasta files of bacterial strains were downloaded with NCBI genome download (https://github.com/kblin/ncbi-genome-download/commit/79b71d3). Using the searchdesc function from the okfasta package (https://github.com/kylebittinger/okfasta/commit/d5736f0), DHFR and TMP-related genes were identified and saved separately. Next, R was used to parse through these DHFR fasta files and to count susceptibility or resistance to TMP based on the annotation provided by RefSeq.

### Statistics

All analysis was performed using GraphPad Prism 9. IC_50_ was calculated by performing nonlinear regression curve fit. No statistical tests were performed.

### Study approval

The purpose of the study was to evaluate the biodistribution and kinetics of [^11^C]-TMP in humans. This study was approved by The University of Pennsylvania IRB (no. 827333). Patients with suspected or confirmed bacterial infections were screened against the inclusion/exclusion criteria (Figure 3 and Supplemental Figure 8) and enrolled after providing written informed consent for a University of Pennsylvania IRB–approved study to evaluate the biodistribution and kinetics of [^11^C]-TMP PET/CT imaging in humans between March 2018 and January 2021 at the Hospital of the University of Pennsylvania. Written informed consent was obtained and recorded for the use of patient images. The study protocol is described at Clinicaltrials.gov (NCT03424525). No power/sample size tests were calculated, and the investigators were not blinded while the data was collected or analyzed. All images and patient data are deidentified. Participant characteristics are provided in Supplemental Figure 8. [^11^C]-TMP was prepared according to good manufacturing practices and as described above at the Penn Cyclotron Facility. Tracer was administered i.v. at doses listed in figure legends, and [^11^C]-TMP PET/CT was acquired on a Phillips Ingenuity PET/CT scanner (43). Images were analyzed using MIM software. SUV_max_ based on the highest uptake voxel within a volume of interest was used to quantitate uptake in amorphous lesions to minimize difficult to assess heterogeneous partial volume effects, while SUV_mean_ based on the mean uptake in typically larger volumes of interest was used to quantify uptake in reference tissues and aortic blood pools where partial volume effects are less of a concern.

## Author contributions

IKL, DAJ, and MAS conceived of the project and experimental design. IKL, DAJ, and OA performed experiments, analyzed data, and interpreted the results. VT and KB designed and performed bioinformatic analysis and interpretation. DD, DH, and DAM referred patients for the clinical studies. JKC, AJY, TD, and RKD analyzed patient scans. JDN, AR, JME, HSL, and RHM were responsible for chemical and radiotracer synthesis. LJG and RMK contributed reagents, analysis, and interpretation. IKL, DAJ, MAS, and JKC wrote the manuscript with input from all authors. IKL and DAJ share co–first authorship, as they equally contributed to the work; the co–first authorship order was assigned based on the length of their involvement and contribution to the study.

## Supplementary Material

Supplemental data

ICMJE disclosure forms

## Figures and Tables

**Table 2:**
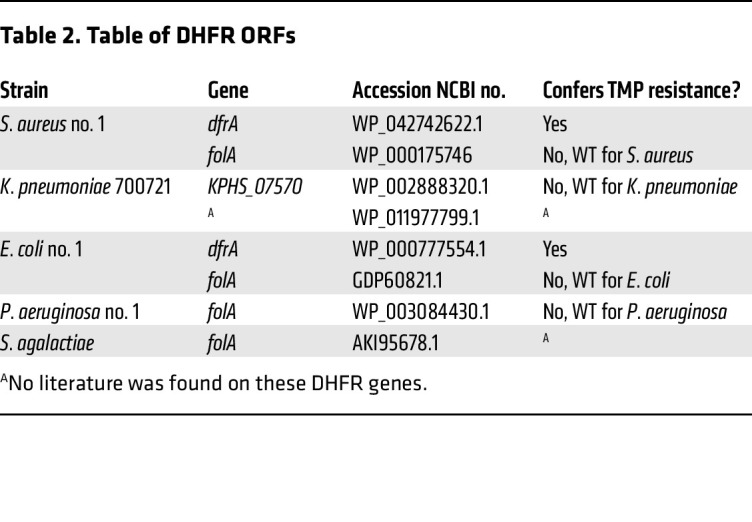
Table of DHFR ORFs

**Table 1:**
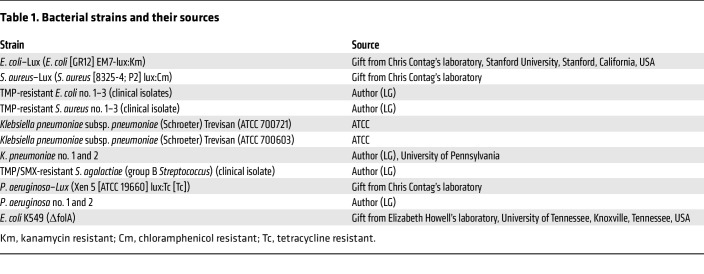
Bacterial strains and their sources

## Version 1 09/15/2022

Electronic publication

## References

[1] Vinjamuri S (1996). Comparison of 99mTc infecton imaging with radiolabelled white-cell imaging in the evaluation of bacterial infection. Lancet.

[2] Tucker AT (2018). Discovery of next-generation antimicrobials through bacterial self-screening of surface-displayed peptide libraries. Cell.

[3] Heuker M (2016). Preclinical studies and prospective clinical applications for bacteria-targeted imaging: the future is bright. Clin Transl Imaging.

[4] Palestro CJ (2007). Diagnostic imaging tests and microbial infections. Cell Microbiol.

[5] Gowrishankar G (2017). Specific imaging of bacterial infection using 6’’-(18)F-fluoromaltotriose: a second-generation PET tracer targeting the maltodextrin transporter in bacteria. J Nucl Med.

[6] Mutch C (2018). [ 11 C]Para-aminobenzoic acid: a positron emission tomography tracer targeting bacteria-specific metabolism. ACS Infect Dis.

[7] Neumann KD (2017). Imaging active infection in vivo using D-amino acid derived PET radiotracers. Sci Rep.

[8] Zhang Z (2018). Positron emission tomography imaging with 2-[(18)F]F- p-aminobenzoic acid detects Staphylococcus aureus infections and monitors drug response. ACS Infect Dis.

[9] Weinstein EA (2014). Imaging Enterobacteriaceae infection in vivo with 18F-fluorodeoxysorbitol positron emission tomography. Sci Transl Med.

[10] Ordonez AA (2021). Imaging Enterobacterales infections in patients using pathogen-specific positron emission tomography. Sci Transl Med.

[11] Ordonez AA (2022). 11C-para-aminobenzoic acid PET imaging of S. aureus and MRSA infection in preclinical models and humans. JCI Insight.

[12] Sellmyer MA (2017). Bacterial infection imaging with [(18)F]fluoropropyl-trimethoprim. Proc Natl Acad Sci U S A.

[13] Sellmyer MA (2017). Quantitative PET reporter gene imaging with [(11)C]trimethoprim. Mol Ther.

[14] Baccanari DP, Kuyper LF (1993). Basis of selectivity of antibacterial diaminopyrimidines. J Chemother.

[15] Ordonez AA (2019). Molecular imaging of bacterial infections: overcoming the barriers to clinical translation. Sci Transl Med.

[16] Huovinen P (1995). Trimethoprim and sulfonamide resistance. Antimicrob Agents Chemother.

[17] Bankevich A (2012). SPAdes: a new genome assembly algorithm and its applications to single-cell sequencing. J Comput Biol.

[18] Parks DH (2015). CheckM: assessing the quality of microbial genomes recovered from isolates, single cells, and metagenomes. Genome Res.

[19] Lombardo MN N GD (2016). Crystal structures of trimethoprim-resistant DfrA1 rationalize potent inhibition by propargyl-linked antifolates. ACS Infect Dis.

[20] Heaslet H (2009). Structural comparison of chromosomal and exogenous dihydrofolate reductase from Staphylococcus aureus in complex with the potent inhibitor trimethoprim. Proteins.

[21] Lamb KM (2014). Crystal structures of Klebsiella pneumoniae dihydrofolate reductase bound to propargyl-linked antifolates reveal features for potency and selectivity. Antimicrob Agents Chemother.

[22] Aeschlimann JR (2003). The role of multidrug efflux pumps in the antibiotic resistance of Pseudomonas aeruginosa and other gram-negative bacteria. Insights from the Society of Infectious Diseases Pharmacists. Pharmacotherapy.

[23] Webber MA, Piddock LJ (2003). The importance of efflux pumps in bacterial antibiotic resistance. J Antimicrob Chemother.

[24] Kohler T (1996). Multidrug efflux in intrinsic resistance to trimethoprim and sulfamethoxazole in Pseudomonas aeruginosa. Antimicrob Agents Chemother.

[26] Weiner-Lastinger LM (2020). Antimicrobial-resistant pathogens associated with adult healthcare-associated infections: summary of data reported to the National Healthcare Safety Network, 2015-2017. Infect Control Hosp Epidemiol.

[27] Rubin RH, Swartz MN (1980). Trimethoprim-sulfamethoxazole. N Engl J Med.

[28] Gleckman R (1981). Trimethoprim: mechanisms of action, antimicrobial activity, bacterial resistance, pharmacokinetics, adverse reactions, and therapeutic indications. Pharmacotherapy.

[29] Phetsang W (2016). Fluorescent trimethoprim conjugate probes to assess drug accumulation in wild type and mutant Escherichia coli. ACS Infect Dis.

[30] Cai H (2009). Development of a liquid chromatography/mass spectrometry-based drug accumulation assay in Pseudomonas aeruginosa. Anal Biochem.

[31] Davis TD (2014). General platform for systematic quantitative evaluation of small-molecule permeability in bacteria. ACS Chem Biol.

[32] Combs MP (2021). Lung microbiota predict chronic rejection in healthy lung transplant recipients: a prospective cohort study. Lancet Respir Med.

[33] Demiroglu H (2018). Radiosynthesis and biodistribution of Tc-99m-trimethoprim: a novel radiolabeled antibiotic for bacterial infection imaging using experimental animals. Kafkas Univ Vet Fak Derg.

[34] Rahmim A, Zaidi H (2008). PET versus SPECT: strengths, limitations and challenges. Nucl Med Commun.

[35] Rashid U (2016). Design, synthesis, antibacterial activity and docking study of some new trimethoprim derivatives. Bioorg Med Chem Lett.

[36] Bolger AM (2014). Trimmomatic: a flexible trimmer for Illumina sequence data. Bioinformatics.

[37] Martin M (2011). Cutadapt removes adapter sequences from high-throughput sequencing reads. EMBnet J.

[38] Li H, Durbin R (2009). Fast and accurate short read alignment with Burrows-Wheeler transform. Bioinformatics.

[39] Eren AM (2015). Anvi’o: an advanced analysis and visualization platform for ‘omics data. PeerJ.

[40] Hyatt D (2010). Prodigal: prokaryotic gene recognition and translation initiation site identification. BMC Bioinformatics.

[41] McArthur AG (2013). The comprehensive antibiotic resistance database. Antimicrob Agents Chemother.

[42] Langmead B, Salzberg SL (2012). Fast gapped-read alignment with Bowtie 2. Nat Methods.

[43] Kolthammer JA (2014). Performance evaluation of the Ingenuity TF PET/CT scanner with a focus on high count-rate conditions. Phys Med Biol.

