# Supplementary material for: Imaging sensitive and drug-resistant bacterial infection with [11C]-trimethoprim
Source: J Clin Invest. 2022 Sep 15;132(18):e156679. doi: 10.1172/JCI156679 (PMC9479701; doi:10.1172/JCI156679)
Supplement: Supplemental data [file jci-132-156679-s112.pdf]

Imaging Sensitive and Drug-Resistant Bacterial Infection with [^{11}C]-Trimethoprim

Iris K. Lee[1,2†], Daniel A. Jacome[1†], Joshua K. Cho[1], Vincent Tu[3], Anthony J. Young[1], Tiffany Dominguez[1], Justin D. Northrup[1], Jean M. Etersque[1,4], Hsiaoju S. Lee[1], Andrew Ruff [1], Ouniol Aklilu[1], Kyle Bittinger[3], Laurel J. Glaser[5], Daniel Dorgan[6], Denis Hadjiliadis[6], Rahul M. Kohli[4,7], Robert H. Mach[1], David A. Mankoff[1], Robert K. Doot[1], and Mark A. Sellmyer[1,4]*

[1]Department of Radiology, University of Pennsylvania, Philadelphia, PA 19104, USA;
[2]Department of Bioengineering, University of Pennsylvania;
[3]Department of Gastroenterology, Hepatology and Nutrition, Children's Hospital of Philadelphia;
[4]Department of Biochemistry and Biophysics, University of Pennsylvania;
[5]Department of Pathology and Laboratory Medicine, Hospital of the University of Pennsylvania;
[6]Department of Medicine, Division of Pulmonary and Critical Care Medicine, University of Pennsylvania
[7]Department of Medicine, Division of Infectious Disease, University of Pennsylvania

† Co-first authorship

* Correspondence should be addressed to:

Mark A. Sellmyer (mark.sellmyer@pennmedicine.upenn.edu)

https://orcid.org/0000-0002-1407-1905

Department of Radiology

Perelman School of Medicine at the University of Pennsylvania

813A Stellar-Chance Labs

422 Curie Boulevard

Philadelphia, PA 19104-6059

Phone: 215-573-3212

Supplemental Figures

A

Strain	Trimethoprim MIC (μg/mL)
E. coli-Lux	1.5
S. aureus-Lux	2
K. pneumoniae 700603	3
K. pneumoniae 700721	>32
K. pneumoniae #1	>32
K. pneumoniae #2	>32
S. aureus #1	>32
S. aureus #2	>32
S. aureus #3	>32
E. coli #1	>32
E. coli #2	>32
E. coli #3	>32
P. aeruginosa #1	>32
P. aeruginosa #2	>32
P. aeruginosa-Lux	>32
S. agalactiae	>32

B

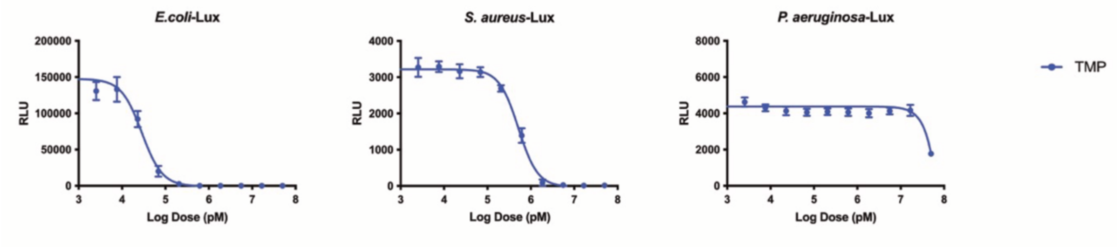

C

Strain	TMP IC_{50} (nM)
E. coli-Lux	66
S. aureus-Lux	950
K. pneumoniae 700603	1200
K. pneumoniae 700721	N/A
K. pneumoniae #1	21000
K. pneumoniae #2	26000
S. aureus #1	N/A
S. aureus #2	N/A
S. aureus #3	N/A
E. coli #1	N/A
E. coli #2	N/A
E. coli #3	N/A
P. aeruginosa #1	N/A
P. aeruginosa #2	N/A
P. aeruginosa-Lux	N/A
S. agalactiae	9100

Figure S1. In vitro characterization of susceptibility and resistance of different bacteria against a panel of common antibiotics. A) Table summarizing TMP MIC values in different bacterial strains. **B)** Luminescent readout of dose response assay with TMP on different lux-operon expressing bacterial strains. **C)** Table summarizing TMP IC_{50} in different bacterial strains. "N/A" denotes ambiguous curve on GraphPad Prism software.

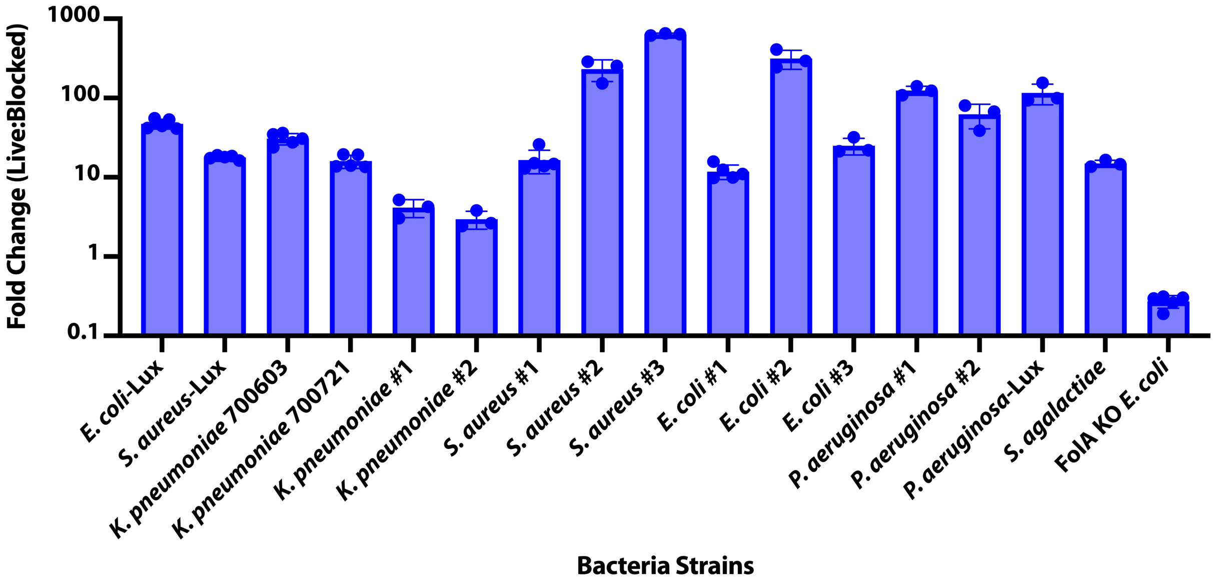

Figure S2. **Representative in vitro [^{11}C]-TMP uptake in bacterial strains**. [^{11}C]-TMP uptake in bacterial cultures after a 30-minute incubation with [^{11}C]-TMP normalized by blocked (50 µM unlabeled TMP) control conditions.

Sample ID	Genome size (bp)	Contigs	Genes (#)	Completeness (%)	Contamination (%)	Strain Heterogeneity	N_{50} (Scaffolds)	GC (%)	Mean Coverage
E. coli	5097133	290	4917	99.56	0.43	0	119500	50.6	38.88
K. pneumoniae 700721	5603052	459	5360	99.71	0.32	11.11	107184	57	29.95
P. aeruginosa	6666409	400	6446	99.54	0.55	11.11	45469	66.2	23.75
S. agalactiae	2073226	84	2095	99.78	0.13	0	63952	35.3	102.3
S. aureus	2781448	157	2617	99.11	0.32	0	50368	32.7	70.55

Figure S3. Completeness CheckM table

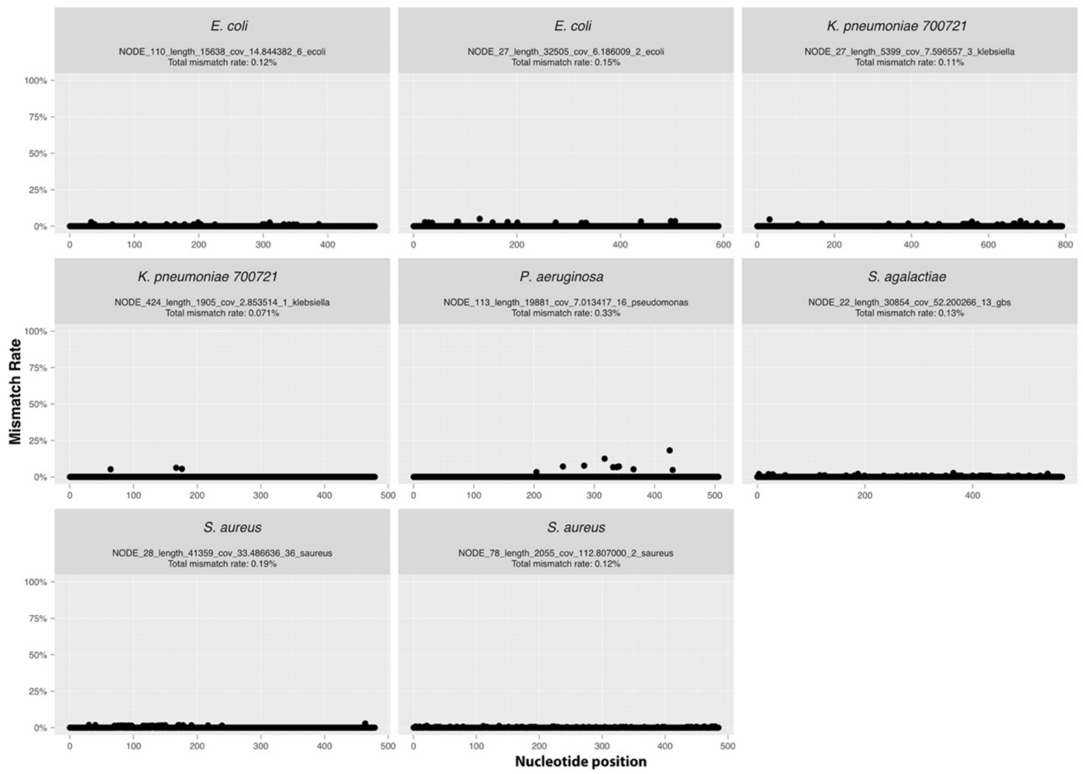

Figure S4. Pile up analysis on DHFR genes from assembled genomes.

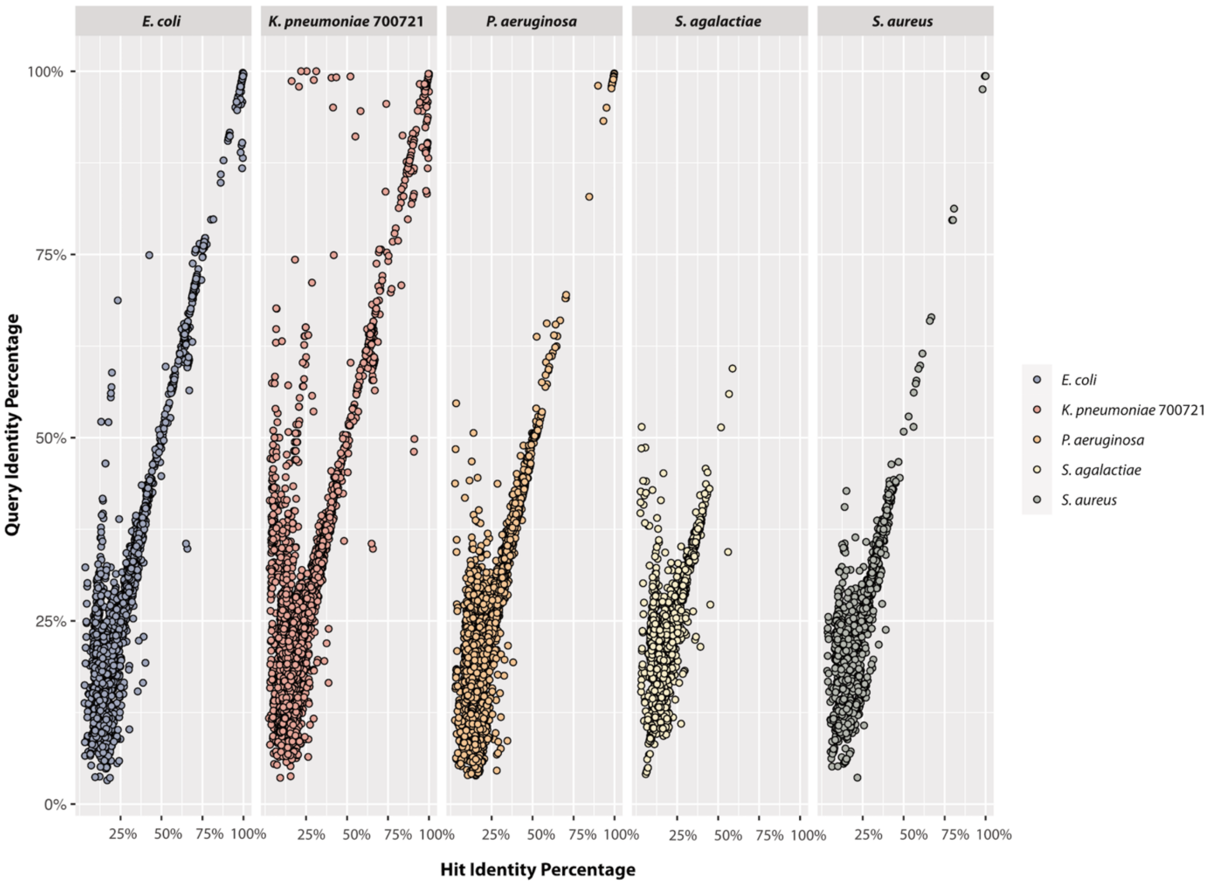

Figure S5. Blastp results relating assembled ORFs from the TMP-resistant subset of bacteria against different previously annotated antibiotic resistance genes in the Comprehensive Antibiotic Resistance Database.

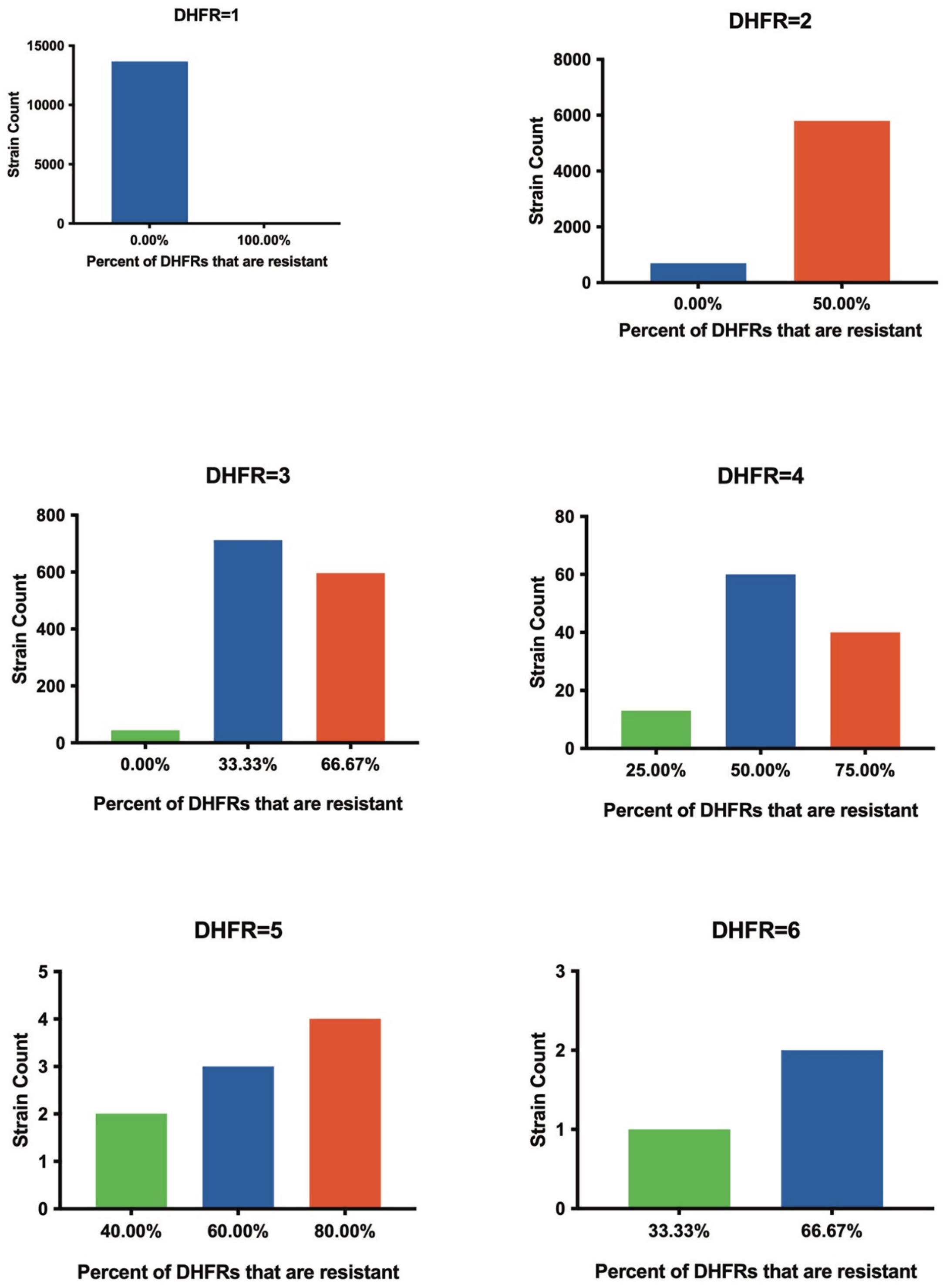

Figure S6. Distribution of resistance conferring DHFR genes based on total DHFR gene number within an *E. coli* strain.

Percent of genes within a species that are resistant (%)

Number of DHFR genes within a strain

Species	0%	25%	33.3%	40%	50%	66.7%	60%	75%	80%	100%	Total
E. coli	**14416**	**13**	**713**	**2**	**5855**	**3**	**598**	**40**	**4**	**11**	**21655**
1	13673									11	13684
2	699				5795						6494
3	44		712				596				1352
4		13			60			40			113
5				2		3			4		9
6			1				2				3
K. pneumoniae	**3597**	**17**	**614**	**4**	**4870**	**5**	**526**	**65**	**2**	**2**	**9702**
1	3101									1	3102
2	465				4804					1	5270
3	26		614				526				1166
4	5	17			66			65			153
5				4		5			2		11
S. aureus	**10403**		**18**		**1610**		**21**			**7**	**12059**
1	10339									7	10346
2	59				1610						1669
3	5		18				21				44

Figure S7. Table showing expected pattern of resistance and DHFR genes within a single bacterial strain for select species. The vertical axis displays the number of DHFR genes carried by the bacterial strain while the horizontal axis shows the percent of those genes carried that are resistant. The data in the central section of the table is the total number of genomes with the corresponding DHFR genes and resistance proportions.

Inclusion Criteria
**1.** At least 18 years of age
**2.** Known or suspected bacterial infection
**3.** Participants must be informed of the investigational nature of this study and provide written informed consent in accordance with institutional and federal guidelines prior to study-specific
**Exclusion Criteria**
**1.** Antibiotic therapy with trimethoprim within 48h of the baseline PET/CT scan
**2.** Inability to tolerate imaging procedures in the opinion of an investigator or treating physician
**3.** Serious or unstable medical or psychological comorbidities that, in the opinion of the investigator, would compromise the subject's safety or successful participation in the study
**4.** Females who are pregnant or breast feeding at the time of screening will not be eligible for this study; a urine pregnancy test will be performed in women of child-bearing potential at screening.

Subject characteristics for [^{11}C]TMP PET scans in dynamic or biodistribution cohorts

ID*	M/F†	Age (y)	Ht‡ (cm)	Wt§ (kg)	IA‖ (MBq)	Underlying disease	Active acute antibiotics	Culture	WBC¶
4	M	64	179	72	563	Lung adenocarcinoma	None	N/A	7.9
6	F	21	172	58	325	Cystic fibrosis	Amikacin, Piperacillin-tazobactam	*S. aureus*#, *P. aeruginosa*#	6.5
13**	F	44	163	52	487	Cystic fibrosis	Only maintenance	*E. coli*#, *Achromobacter*#	13.2
13**	F	44	163	59	780	Cystic fibrosis	Ceftriaxone	*S. aureus*#, *Achromobacter*#	8.3
14&	M	55	168	79	672	Discitis/osteomyelitis	Cefazolin	*S. aureus* (MSSA)@	7.6
12&	M	54	168	84	129	Discitis/osteomyelitis	Vancomycin	*S. aureus* (MSSA)@	7.8

*Identification number for consented patients who successfully completed [^{11}C]TMP imaging session.
†Male or female gender.
‡ Height (cm).
§Weight (kg).
‖Injected [^{11}C]TMP activity (MBq).
¶White blood cell count ($\times 10^9$ cells per liter).
#Sputum culture source.
**Subject 13's characteristics for second [^{11}C]TMP scan listed in row immediately after row with first scan characteristics
&Subject 12 in the biodistribution cohort was reconsented as subject 14 in the dynamic cohort.
@Aspirate culture source.

Figure S8. Inclusion/exclusion criteria and demographics table of subject characteristics for [^{11}C]-TMP trial diagram.

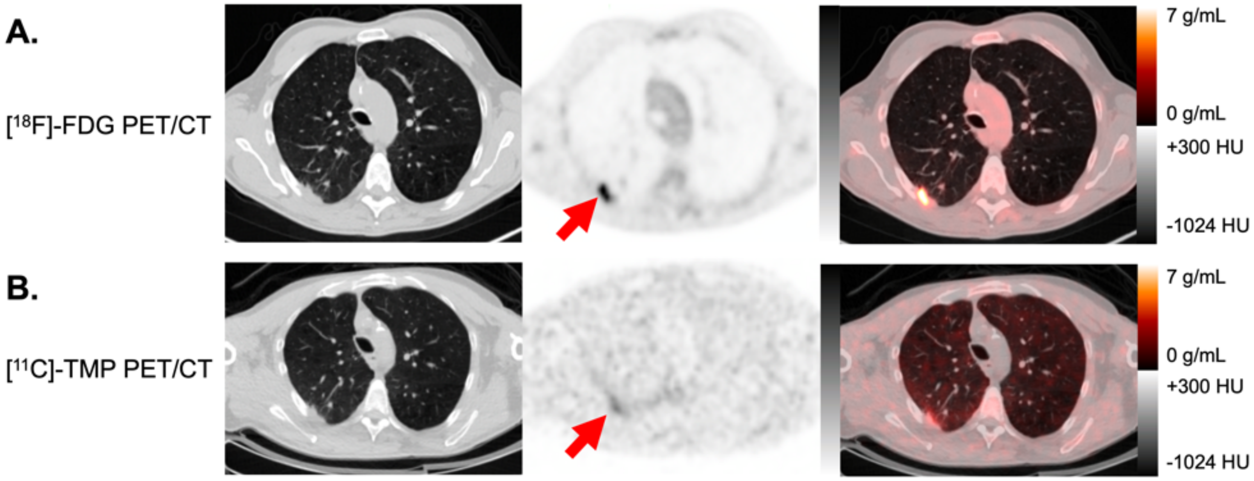

Figure S9. A) [18F]-FDG and **B**) [11C]-TMP uptake in cancer in 64-year-old man with known lung adenocarcinoma seen in Figure 4A. PET images are scaled 0-7 g/mL SUV are CT images are scaled -1024 to +300 HU.

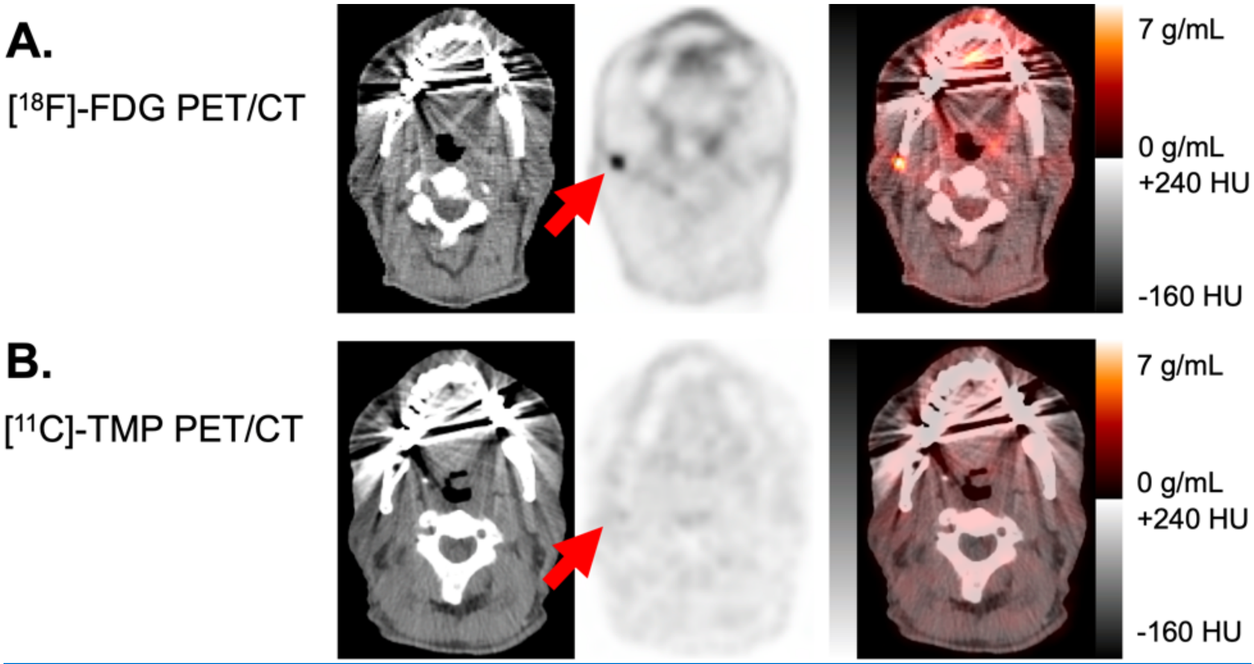

Figure S10. A) [18F]-FDG and **B**) [11C]-TMP uptake in suspected lung cancer metastasis in the neck of a 64-year-old man with known lung adenocarcinoma seen in Figure 4A. PET images are scaled 0-7 g/mL SUV and CT images are scaled -160 to +240 HU.

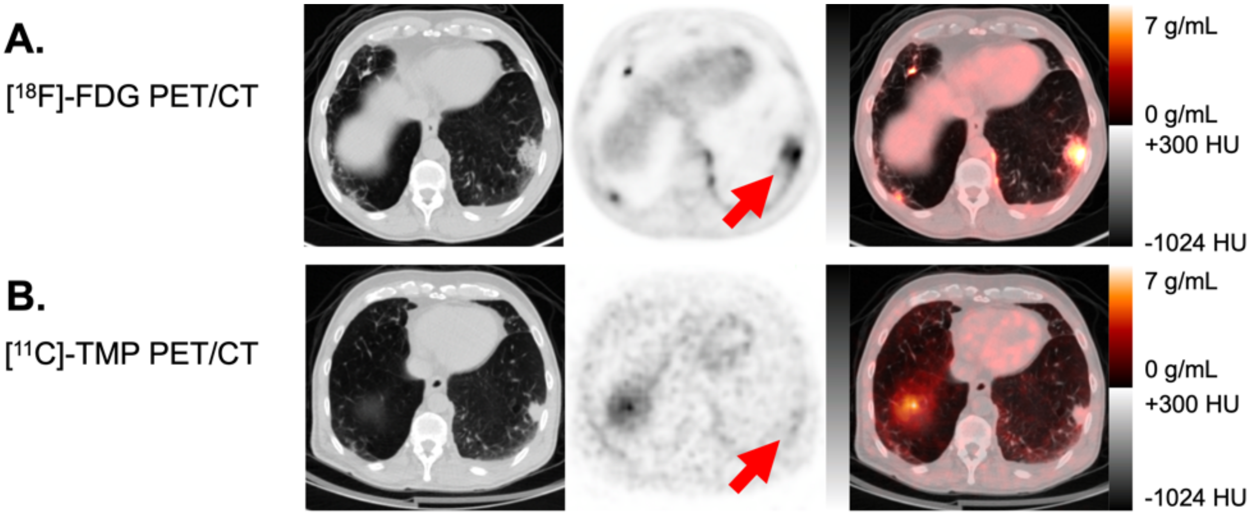

Figure S11. A) [^{18}F]-FDG and **B**) [^{11}C]-TMP uptake in suspected sterile lung pneumonitis in 64-year-old man seen in Figure 4A. Resolution of pneumonitis was observed after 2 days in [^{11}C]-TMP PET/CT, with a decrease in CT volume and increase in CT enhancement. PET images are scaled 0-7 g/mL SUV and CT images are scaled -1024 to +300 HU.

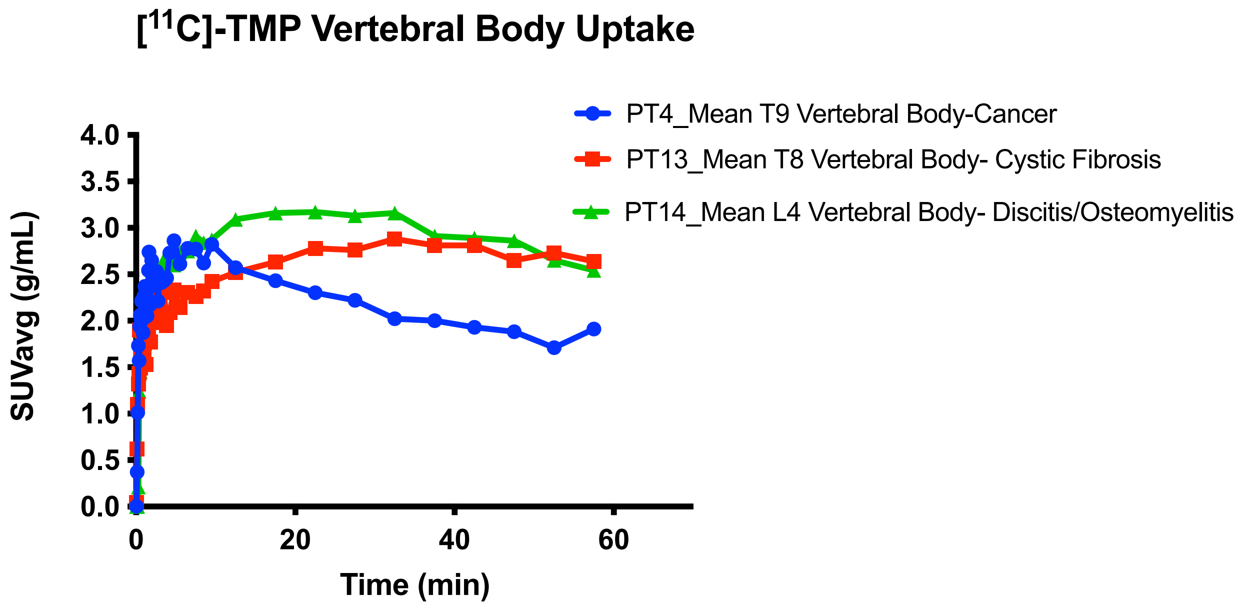

Figure S12. Vertebral body uptake as a function of time.

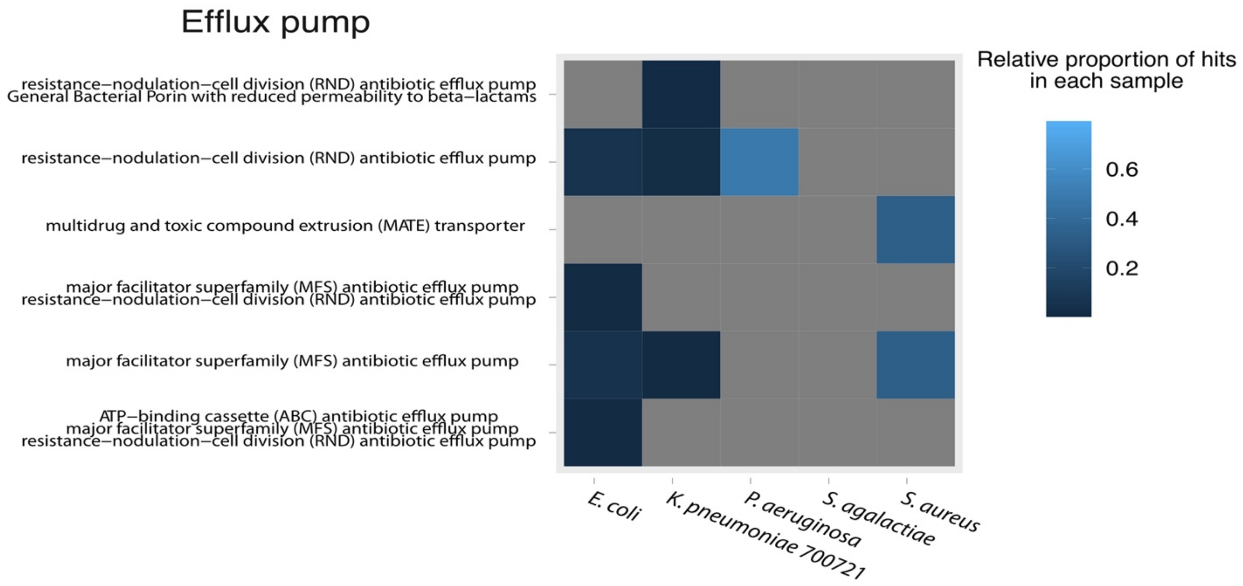

Figure S13. Heatmap of efflux pump genes expressed in the panel of TMP-resistant bacteria. Relative proportion is relative to the total number of genes within the species genome that had greater than 90% homology to Comprehensive Antibiotic Resistance Database annotated genes.

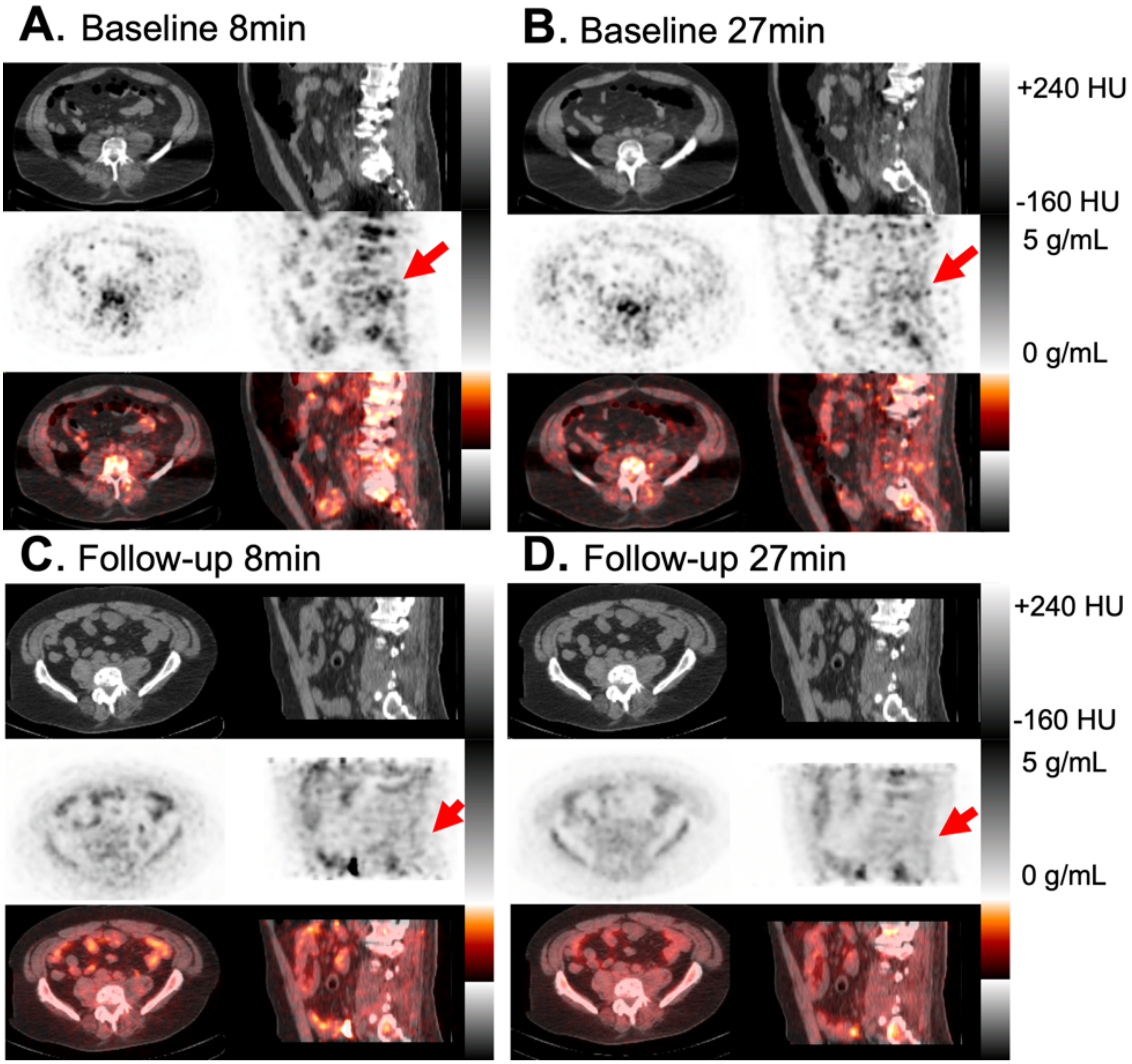

Figure S14. Baseline (**A** and **B**) and follow-up (**C** and **D**) [^{11}C]-TMP PET/CT in 55 year-old man with biopsy proven discitis-osteomyelitis seen in Figure 7. Images acquired 8 and 27 minutes post-injection (P.I.). PET images are scaled 0-5 g/mL SUV and CT images are scaled -160 to +240 HU.
